# The clinical spectrum of MECP2 variants in males: a multicenter Turkish cohort of 22 probands, with implications for diagnosis and variant interpretation

**DOI:** 10.1186/s12887-026-07905-0

**Published:** 2026-10-21

**Authors:** Çağrı Doğan, Mehmet Burak Mutlu, Ali Cansu, Esra Habiloğlu, Murat Öztürk, Uğur Gümüş, Abdullah İhsan Gürler, Bahtiyar Şahinoğlu, Hamdi Kale, Mustafa Tarık Alay, Abdullah Sezer, Aysel Kalaycı, Elif Yılmaz Güleç, Süheyla Emre, Hamza Polat, Zehra Manav Yiğit, Hilmi Bolat, Zeynep Esener, Dilan Genç Akdağ, Gül Ünsel Bolat, Ayberk Türkyılmaz, Akif Ayaz, Abdulkerim Kolkıran, Ahmet Cevdet Ceylan, Taha Solmaz, Yusuf Bahap, Neslihan Cinkara, Sümeyra Oğuz, Elif Uzay, Safiye Güneş Sager, Alper Han Çebi, Oğuzhan Demir, Ezgi Susam, İrem Kalay

**Affiliations:** 1 Department of Medical Genetics, Ordu University Training and Research Hospital, Ordu, Türkiye Turkey; 2 Detagen Genetic Diagnosis Center, Kayseri, Türkiye Turkey; 3 Department of Pediatric Neurology, Faculty of Medicine, Karadeniz Technical University, Trabzon, Türkiye Turkey; 4 Department of Medical Genetics, Batman Training and Research Hospital, Batman, Türkiye Turkey; 5 Department of Medical Genetics, Dr. Ersin Arslan Training and Research Hospital, Gaziantep, Türkiye Turkey; 6 Department of Medical Genetics, Ankara Etlik City Hospital, Ankara, Türkiye Turkey; 7 Department of Medical Genetics, Cerrahpaşa University Hospital, İstanbul, Türkiye; 8 Department of Medical Genetics, Medeniyet University Hospital, İstanbul, Türkiye; 9 Department of Medical Genetics, Gazi Yaşargil Training and Research Hospital, Diyarbakır, Türkiye Turkey; 10 Department of Medical Genetics, Adnan Menderes University Hospital, Aydın, Türkiye Turkey; 11 Department of Medical Genetics, Balıkesir University Hospital, Balıkesir, Türkiye Turkey; 12 Department of Child and Adolescent Psychiatry, Balıkesir University Hospital, Balıkesir, Türkiye Turkey; 13 Gelişim Medical Diagnostic Center, İstanbul, Türkiye; 14 Genetic Diseases Diagnosis and Evaluation Center, İstinye University, İstanbul, Türkiye; 15 Department of Pediatric Genetics, Ankara Etlik City Hospital, Ankara, Türkiye Turkey; 16 Department of Medical Genetics, Ankara Bilkent City Hospital, Ankara, Türkiye Turkey; 17 Department of Medical Genetics, Gazi University, Ankara, Türkiye Turkey; 18 Department of Medical Genetics, Trabzon Kanuni Training and Research Hospital, Trabzon, Türkiye Turkey; 19 Department of Medical Genetics, Zeynep Kamil Women’s and Children’s Diseases Training and Research Hospital, İstanbul, Türkiye; 20 Hamidiye International School of Medicine, Kartal Dr. Lütfi Kırdar City Hospital, University of Health Sciences Türkiye, İstanbul, Türkiye; 21 Department of Medical Genetics, Faculty of Medicine, Karadeniz Technical University, Trabzon, Türkiye Turkey; 22 Ankara Bilkent City Hospital, Health Institutes of Türkiye (TÜSEB), Ankara, Türkiye Turkey; 23 Department of Medical Genetics, University of Health Sciences Sisli Hamidiye Etfal Training and Research Hospital, Istanbul, Turkey

**Keywords:** MECP2, Males, Hemizygous, Rett syndrome, Variant classification, ClinGen, Intellectual disability, Epilepsy

## Abstract

**Background:**

In males, MECP2 loss of function was long thought to be lethal or uniformly severe, and affected boys remain under-recognised. Their clinical range is still poorly defined.

**Methods:**

We assembled a multicentre Turkish series of 22 hemizygous male probands ascertained through routine diagnostic testing rather than severe-phenotype registries. Variants were classified under the ClinGen Expert Panel specification for MECP2 (GN036, version 6.0.0) and its combining rules, with the Panel’s ClinVar classifications adopted where available. Phenotypic analysis was restricted to the 21 probands with pathogenic, likely pathogenic or uncertain variants.

**Results:**

The 22 probands carried 17 distinct variants (11 missense, 5 frameshift and one 3’ untranslated region deletion), of which 2 were classified as pathogenic, 1 likely pathogenic, 13 uncertain and 1 benign (3, 4, 14 and 1 probands, respectively). Where recorded, intellectual disability or developmental delay affected 7 of 7 probands with pathogenic or likely pathogenic variants and 8 of 10 with uncertain variants; seizures affected 2 of 3 and 8 of 10, respectively. Growth or head-circumference findings, recorded in 9 of 21 probands, included obesity or overgrowth (5), microcephaly (4, one borderline) and short stature (3). A maternal carrier was identified in 6 of 22 families (five laboratory-confirmed); the other 16 mothers were untested, so no de novo event was confirmed. Second-locus findings, reported by referring laboratories in 3 probands, could not be independently reclassified.

**Conclusions:**

In this diagnostically ascertained series, phenotypes observed among males carrying MECP2 variants were graded rather than uniformly severe. Fourteen of the 21 probands carry variants of uncertain significance, in whom causation is not established, so the range that may be attributed to MECP2 rests on seven individuals carrying variants classified as pathogenic or likely pathogenic under the stated criteria. In one of them, published functional data on the identical variant conflict with its pathogenic classification, so attribution of his phenotype to MECP2 remains uncertain. These provisional observations support a low threshold for MECP2 testing in males with unexplained neurodevelopmental presentations, attention to growth as well as head circumference, and consideration of additional loci.

**Supplementary Information:**

The online version contains supplementary material available at https://doi.org/10.1186/s12887-026-07905-0.

## Background

 MECP2 encodes methyl-CpG-binding protein 2, a chromatin-associated protein that reads methylated DNA and recruits transcriptional corepressors [1, 2]. The gene is subject to X inactivation and produces two principal isoforms through alternative splicing of exon 2, MeCP2_e1 and MeCP2_e2, of which MeCP2_e1 predominates in brain [3, 4]. MeCP2 binds methylated cytosine through its methyl-CpG-binding domain and represses transcription through an interaction with the NCoR/SMRT corepressor complex, an arrangement in which the NCoR interaction domain within the transcriptional repression domain is central [2, 5]. The bridging function of the protein, rather than any single catalytic activity, appears to underlie its dosage sensitivity [5, 6].

Heterozygous loss-of-function variants in females cause Rett syndrome [4], and duplication of the locus in males causes MECP2 duplication syndrome. Hemizygous loss of function in males was for many years considered lethal in the neonatal period or uniformly severe, and the classic teaching held that surviving males were rare and encephalopathic [7]. That view has been progressively revised. Series of hemizygous males have described presentations ranging from severe infantile encephalopathy to milder syndromic intellectual disability and to phenotypes dominated by epilepsy or by autistic features [8–11]. Reports of PPM-X syndrome, of the recurrent hypomorphic allele p.Ala152Val and of further familial and sporadic male cases have widened the recognised range [12–15].

Two developments make a reappraisal timely. First, the mechanistic basis of C-terminal truncating variants has become clearer. Work on frameshift variants in the C-terminal domain has indicated that the consequence depends not only on the position of the deletion but on the translational reading frame that results, since different frames produce different downstream peptides before termination [16, 17]. Second, variant interpretation in MECP2 has been placed on a formal footing. The ClinGen Rett and Angelman-like Disorders Variant Curation Expert Panel has published a gene-specific specification of the ACMG/AMP framework [18–20], and the interpretation of predicted loss-of-function variants has been standardised through a decision tree that weighs transcript position and the proportion of protein lost [21]. Population reference data have likewise improved, with gnomAD v4 providing hemizygote counts on the X chromosome at a scale not previously available [22].

Most published male series have been ascertained through Rett syndrome registries or through severe-phenotype cohorts, which selects against the milder end of the range. We therefore assembled a series ascertained through routine diagnostic genetic testing across Türkiye, in which males were tested for unexplained neurodevelopmental presentations rather than for a suspected MECP2 disorder. Our aims were to describe the clinical range captured by such ascertainment, to reclassify every variant under a single current specification, and to examine what the series does and does not support with respect to variant interpretation.

## Methods

### Ethics approval and consent

**Fig. 1:**
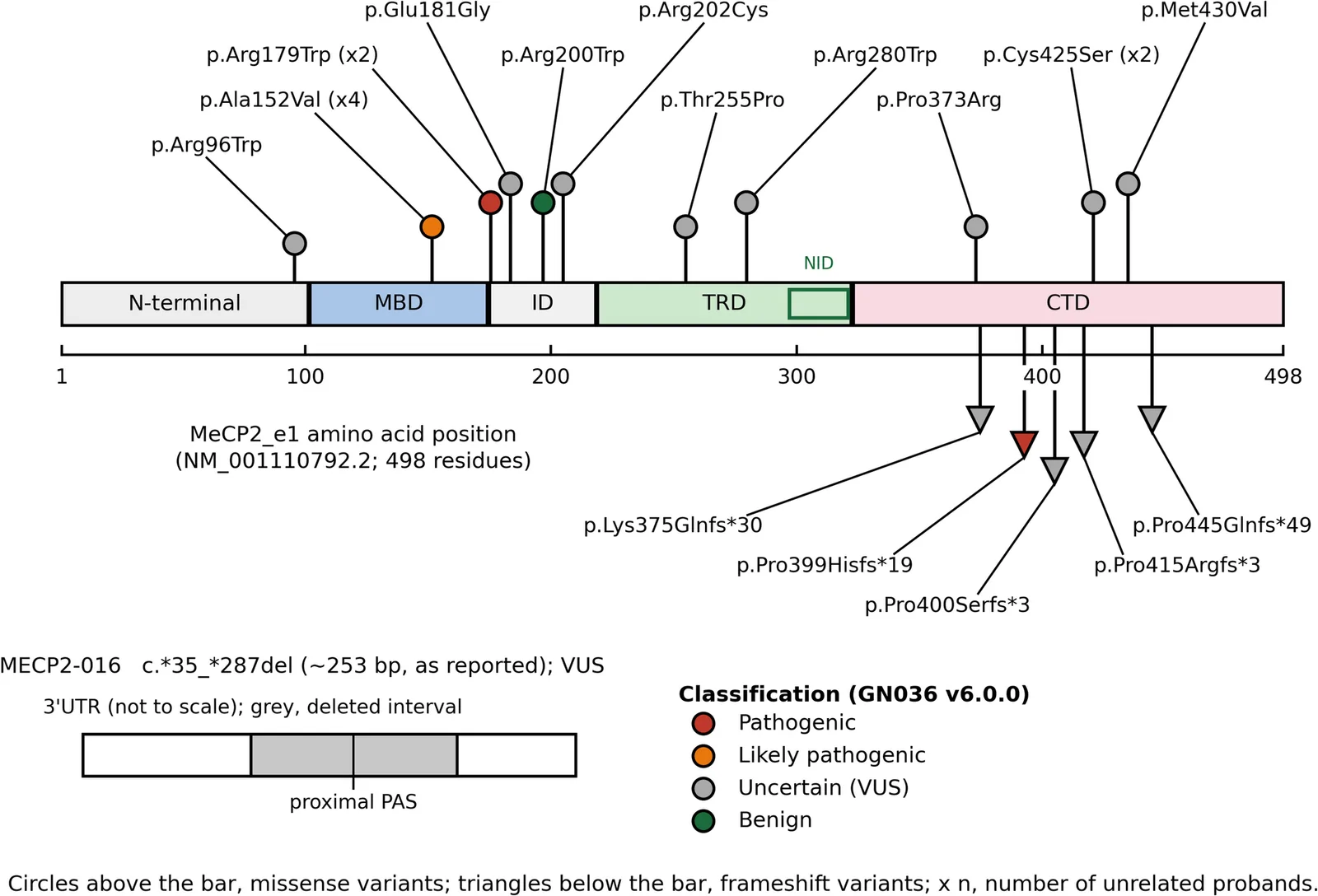
Spectrum of MECP2 variants in the series. The full MeCP2_e1 protein (498 residues, NM_001110792.2) is shown with its domain structure: the methyl-CpG-binding domain (e1 residues 102 to 174), the intervening domain, the transcriptional repression domain (e1 219 to 322) containing the NCoR/SMRT interaction domain (e1 297 to 321, shown as an inner box), and the C-terminal domain; boundaries follow UniProt P51608 and the literature as set out in the Methods, converted to e1 numbering. Missense variants are shown as lollipops above the protein bar, staggered where positions are adjacent, and frameshift variants below it; every marker is labelled, and no unlabelled marks appear on the bar. Recurrent variants are annotated with the number of unrelated probands, and final classifications are colour-coded as shown in the key. The five frameshift variants lie within the large final exon of MeCP2_e1, which encodes the C-terminal domain and is expected to escape nonsense-mediated decay; the region contains short repeat sequences that favour misalignment by replication slippage. The deletion in MECP2-016 lies entirely within the 3’ untranslated region and removes no coding nucleotide; it cannot be placed on the protein axis and is shown on a separate 3’ untranslated region bar drawn beneath the protein and not to scale, with the position of the proximal polyadenylation signal marked within it. MeCP2_e2 (NM_004992.4, MANE Plus Clinical) equivalents for every variant are given in Table 1

The study was performed in accordance with the Declaration of Helsinki and was approved by the Clinical Research Ethics Committee of Ordu University (approval no. 2026/19, dated 23 January 2026). Written informed consent to participate and for publication of de-identified data was obtained from all patients or, for those under 18 years of age, from their parents or legal guardians. For the proband who died in 2025 (MECP2-022), written informed consent had been obtained from his family before his death. The unaffected adult relatives whose genotypes are shown in Fig. 1 provided written informed consent for testing and for publication of their de-identified results. The consent obtained does not extend to the publication of identifiable images, and no such images are included.

### Ascertainment and testing indications

Probands were identified retrospectively from the diagnostic genetic testing records of participating medical genetics centres across Türkiye. Testing was not offered to all patients attending these clinics. Exome sequencing or targeted analysis was requested for defined indications: unexplained developmental delay or intellectual disability, including intellectual disability with prominent neuropsychiatric features and, in one proband born at 25 weeks of gestation, neurodevelopmental impairment judged not to be explained by prematurity alone; refractory or unexplained epilepsy; developmental regression; developmental delay accompanied by dysmorphism; adult-onset unexplained neurological decline in one proband; congenital absence of teeth in one proband; and, in a small number of relatives, family history following the identification of a variant in a proband. The indication recorded for each proband is given in Table 3. Relatives shown in Fig. 1b were tested at the family’s request after genetic counselling and not because of symptoms. Inclusion required male sex, hemizygosity for a MECP2 variant, and availability of clinical records sufficient for the fields reported here.

### Molecular analysis

Sequencing was performed at the referring centres as part of diagnostic care, predominantly by whole-exome sequencing and in some cases by targeted panel analysis. Variant calling, filtering and prioritisation were carried out locally under each laboratory’s own protocol; the diagnostic reports were the source documents for this study. The deletion in MECP2-016 was identified by exome-based copy number analysis at the referring centre; the copy number calling pipeline was not recorded in the diagnostic report, and the finding has not been confirmed by an independent method. The breakpoint coordinates for this deletion are reproduced as stated in the referring laboratory’s report; because the calling pipeline is not recorded and read-depth copy number analysis of exome data cannot by itself resolve an event of this size to single-base precision, we treat these coordinates, and the derived length of 253 base pairs, as approximate (see Results). Chromosome analysis and microarray were normal in that proband, but an event of this size lies below the resolution of microarray and these results therefore exclude a larger rearrangement rather than confirm the deletion. Segregation testing, where performed, was carried out by Sanger sequencing at the referring centres and reported in the diagnostic records. Runs of homozygosity were not analysed, and no systematic search for a second recessive diagnosis beyond each laboratory’s diagnostic pipeline was performed (see Limitations).

### Variant nomenclature and coordinates

**Fig. 2:**
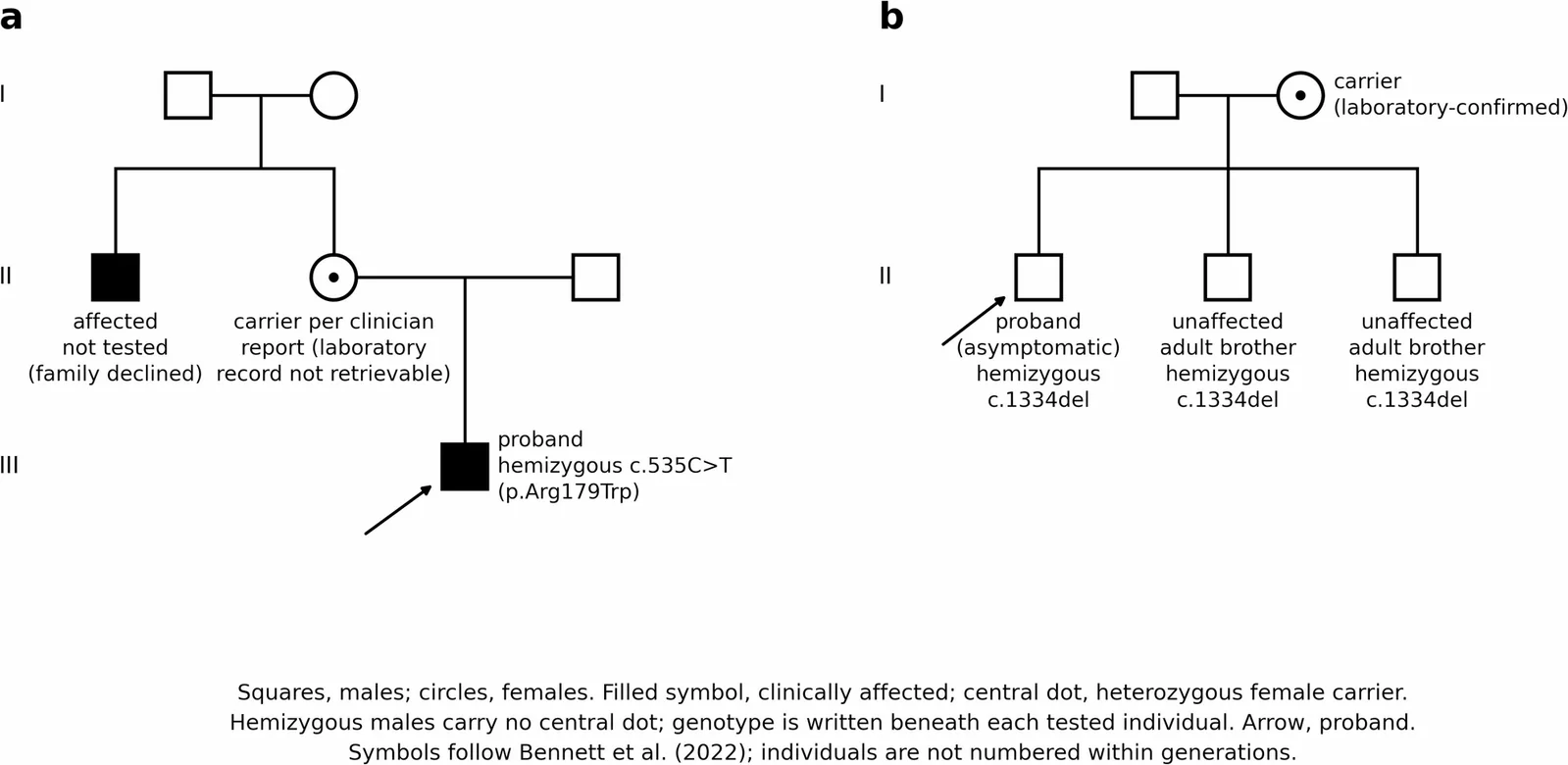
Pedigrees of the two families with genotyped relatives. **a** The family of MECP2-021 (p.Arg179Trp). The mother is a carrier according to the referring clinician’s documentation; the laboratory record was not retrievable. The affected maternal uncle was not genotyped because the family declined testing, so co-segregation is not demonstrated and PP1 was not applied; the pedigree pattern is consistent with X-linked inheritance. The parents are not consanguineous. **b** The family of MECP2-019 (c.1334del). The mother is a laboratory-confirmed carrier. The proband and his two adult brothers are all hemizygous for the variant; all three are clinically unaffected. BS2 at Strong rests on the two unaffected adult brothers; the asymptomatic proband himself is not counted in that evidence. Symbols follow Bennett et al. [25]: squares, males; circles, females; filled symbols, clinically affected; a central dot, heterozygous female carrier. Hemizygous males are not marked with a central dot, since their genotype is written beneath the symbol, as it is for every tested individual. The proband in each family is indicated by an arrow. Individuals are not numbered within generations

All variants were standardised to GRCh38 and are reported primarily against the brain-predominant MeCP2_e1 transcript, NM_001110792.2, which is the MANE Select transcript for MECP2. The MeCP2_e2 transcript, NM_004992.4, carries the MANE Plus Clinical designation. The Expert Panel specification numbers its residue-based provisions on the e2 transcript, so those provisions are cited in e2 numbering throughout; this concerns residue numbering only and is separate from the MANE designations. To prevent misreading, Table 1 gives both nomenclatures for every variant. The transcripts differ by 36 coding nucleotides and 12 residues, so that e2 cDNA numbering equals e1 numbering minus 36 and e2 protein numbering equals e1 numbering minus 12. Variants in the 3’ untranslated region are numbered from the stop codon and are therefore identical in both nomenclatures. Variants originally reported on GRCh37 were converted to GRCh38, and all GRCh38 coordinates in Additional file 1 (Table S1a) were verified against the transcript structure and, for insertions and deletions, normalised with the Ensembl Variant Recoder. Domain boundaries used in Table 1; Fig. 2 are given below in both nomenclatures, as e2 residues followed by their e1 equivalents (e1 equals e2 plus 12): methyl-CpG-binding domain, e2 90 to 162 (e1 102 to 174; UniProt P51608, coinciding with the specification’s PM1 range); intervening domain, e2 163 to 206 (e1 175 to 218); transcriptional repression domain, e2 207 to 310 (e1 219 to 322) [1, 5]; NCoR/SMRT interaction domain, e2 285 to 309 (e1 297 to 321; UniProt P51608), containing the specification’s PM1 hotspot at e2 302 to 306 (e1 314 to 318); and C-terminal domain, e2 311 to 486 (e1 323 to 498). Nuclear localisation signal boundaries are reported inconsistently in the literature and no classification criterion in this study depends on them, so NLS labels are not used.

**Table 1:** MECP2 variants in the 22 male probands, with gnomAD v4.1.0 frequencies, applied ClinGen GN036 v6.0.0 criteria and the combining rule met

Proband	cDNA (e1, NM_001110792.2, MANE Select)	Protein (e1)	e2 (NM_004992.4, MANE Plus Clinical)	Domain	Type	gnomAD v4.1.0 (AC; hemi; AN)	ACMG/VCEP criteria (strength)	Rule met / basis	Class
MECP2-001	c.535 C > T	p.Arg179Trp	c.499 C > T / p.Arg167Trp	Intervening domain	Missense	AC 3; 0 hemi; AN 1,098,208	Expert Panel: PS2_Very Strong, PP1_Strong, PM2_Supporting. This series: 2 unrelated probands (PS4 not applicable: present in gnomAD); PP3	Expert Panel classification adopted (ClinVar 143603)	P
MECP2-002	c.1274G > C	p.Cys425Ser	c.1238G > C / p.Cys413Ser	CTD	Missense	Absent (0)	PM2_Supporting; PS4_Supporting (with 018)	2 Supporting: no rule met	VUS
MECP2-003	c.1243_1252del	p.Pro415Argfs*3	c.1207_1216del / p.Pro403Argfs*3	CTD (last exon)	Frameshift	Absent (0)	PVS1; PM2_Supporting	1 Very Strong + 1 Supporting: no rule met	VUS
MECP2-004	c.1194_1203del	p.Pro399Hisfs*19	c.1158_1167del / p.Pro387Hisfs*19	CTD (last exon)	Frameshift	Absent (0)	PVS1; PS4_Supporting (this proband and the published observation of the identical variant [23, 24]); PM2_Supporting	1 Very Strong + 2 Supporting: pathogenic	P
MECP2-005	c.838 C > T	p.Arg280Trp	c.802 C > T / p.Arg268Trp	TRD	Missense	Exome AC 26; 3 hemi; AN 1,098,176. Genome AC 4; 1 hemi; AN 112,533	Expert Panel: BS2, BP5, PP3	Expert Panel classification adopted (ClinVar 143700)	VUS
MECP2-006	c.763 A > C	p.Thr255Pro	c.727 A > C / p.Thr243Pro	TRD	Missense	Absent (0)	PM2_Supporting	1 Supporting: no rule met	VUS
MECP2-007	c.1118 C > G	p.Pro373Arg	c.1082 C > G / p.Pro361Arg	CTD	Missense	AC 1; 1 hemi; AN 1,097,470	(none applied)	No rule met	VUS
MECP2-008	c.598 C > T	p.Arg200Trp	c.562 C > T / p.Arg188Trp	Intervening domain	Missense	Exome AC 8; 2 hemi; AN 1,098,231. Genome AC 1; 0 hemi; AN 112,215	Expert Panel: BS1, BS2	Expert Panel classification adopted (ClinVar 566728)	B
MECP2-009	c.542 A > G	p.Glu181Gly	c.506 A > G / p.Glu169Gly	Intervening domain	Missense	AC 1; 0 hemi; AN 1,098,250	(none applied)	No rule met	VUS
MECP2-010	c.286 C > T	p.Arg96Trp	c.250 C > T / p.Arg84Trp	N-terminal (MBD-proximal)	Missense	AC 2; 0 hemi; AN 1,097,327	(none applied; e2 residue 84 lies outside the PM1 range of 90 to 162)	No rule met	VUS
MECP2-011	c.455 C > T	p.Ala152Val	c.419 C > T / p.Ala140Val	MBD	Missense	AC 2; 1 hemi; AN 1,097,434	PM1; PP1_Strong (two published families [12, 15]); PP3. PS4 not applicable (PM2 not met)	1 Strong + 1 Moderate: likely pathogenic	LP
MECP2-012	c.455 C > T	p.Ala152Val	c.419 C > T / p.Ala140Val	MBD	Missense	AC 2; 1 hemi; AN 1,097,434	PM1; PP1_Strong (shared); PP3	1 Strong + 1 Moderate: likely pathogenic	LP
MECP2-013	c.455 C > T	p.Ala152Val	c.419 C > T / p.Ala140Val	MBD	Missense	AC 2; 1 hemi; AN 1,097,434	PM1; PP1_Strong (shared); PP3	1 Strong + 1 Moderate: likely pathogenic	LP
MECP2-014	c.604 C > T	p.Arg202Cys	c.568 C > T / p.Arg190Cys	Intervening domain	Missense	AC 8; 2 hemi; AN 1,098,224	Expert Panel: BS2, PS3_Supporting	Expert Panel classification adopted (ClinVar 156667)	VUS
MECP2-015	c.1122dup	p.Lys375Glnfs*30	c.1086dup / p.Lys363Glnfs*30	CTD (last exon)	Frameshift	Absent (0; AN 1,097,238 at the site)	PVS1; PM2_Supporting (PS4 not applied: no external ClinVar submission documents a phenotyped, unrelated affected individual)	1 Very Strong + 1 Supporting: no rule met	VUS
MECP2-016	c.*35_*287del	(3’UTR; no protein change)	c.*35_*287del / (3’UTR; no protein change)	3’UTR (non-coding)	3’UTR deletion, ~ 253 bp (as reported)	n/a	(no applicable code)	No rule met	VUS
MECP2-017	c.1288 A > G	p.Met430Val	c.1252 A > G / p.Met418Val	CTD	Missense	AC 2; 1 hemi; AN 1,096,805	(none applied)	No rule met	VUS
MECP2-018	c.1274G > C	p.Cys425Ser	c.1238G > C / p.Cys413Ser	CTD	Missense	Absent (0)	PM2_Supporting; PS4_Supporting (with 002)	2 Supporting: no rule met	VUS
MECP2-019	c.1334del	p.Pro445Glnfs*49	c.1298del / p.Pro433Glnfs*49	CTD (last exon, very distal)	Frameshift	Absent (0)	PVS1 (first altered residue e2 Pro433, proximal to p.Glu472); PM2_Supporting; BS2 (Strong: 2 phenotyped unaffected hemizygous brothers)	Pathogenic and benign criteria conflict: uncertain	VUS
MECP2-020	c.455 C > T	p.Ala152Val	c.419 C > T / p.Ala140Val	MBD	Missense	AC 2; 1 hemi; AN 1,097,434	PM1; PP1_Strong (shared); PP3	1 Strong + 1 Moderate: likely pathogenic	LP
MECP2-021	c.535 C > T	p.Arg179Trp	c.499 C > T / p.Arg167Trp	Intervening domain	Missense	AC 3; 0 hemi; AN 1,098,208	Expert Panel: PS2_Very Strong, PP1_Strong, PM2_Supporting. This series: 2 unrelated probands (PS4 not applicable: present in gnomAD); PP3. PP1 not applied here (uncle not genotyped)	Expert Panel classification adopted (ClinVar 143603)	P
MECP2-022	c.1198_1202del	p.Pro400Serfs*3	c.1162_1166del / p.Pro388Serfs*3	CTD (last exon)	Frameshift	AC 1; 0 hemi; AN 535,637 (low-coverage site)	PVS1	1 Very Strong alone: no rule met	VUS

All probands are hemizygous males. Variants are reported against MeCP2_e1 (NM_001110792.2, MANE Select) with MeCP2_e2 (NM_004992.4, MANE Plus Clinical) equivalents; the Expert Panel specification numbers its residue-based rules on e2; e2 cDNA = e1 minus 36 and e2 protein = e1 minus 12. 3’ untranslated region variants are numbered from the stop codon and are identical in both nomenclatures. Coordinates were standardised to GRCh38 (Additional file 1, Table S1a, including normalised coordinates for insertions and deletions); the variant in MECP2-020 was converted from GRCh37. Domain boundaries (e2 numbering, e1 = e2 + 12): MBD 90 to 162 (UniProt P51608, coinciding with the specification’s PM1 range); intervening domain 163 to 206; TRD 207 to 310; NID 285 to 309, containing the specification’s PM1 hotspot at 302 to 306; CTD 311 to 486. gnomAD values are exome data from v4.1.0 unless labelled genome; AC, allele count; hemi, hemizygote count; AN, allele number; group-maximum filtering allele frequencies are given in Additional file 1. For variants shown as absent, the surrounding exon carries full allele numbers (approximately 1.10 million alleles), consistent with a covered, variant-free site. Where the class is taken from the Expert Panel, the criteria quoted are those cited in the Panel’s ClinVar submission. P, pathogenic; LP, likely pathogenic; VUS, variant of uncertain significance; B, benign; MBD, methyl-CpG-binding domain; TRD, transcriptional repression domain; NID, NCoR/SMRT interaction domain; CTD, C-terminal domain. Distribution across the 17 distinct variants: 2 P, 1 LP, 13 VUS, 1 B; at proband level across the 22 probands: 3 P, 4 LP, 14 VUS, 1 B

### Variant classification

Every variant was classified independently for this study by a single team, irrespective of the class assigned in the original diagnostic report. Classification followed the ACMG/AMP framework [18] as specified for MECP2 by the ClinGen Rett and Angelman-like Disorders Variant Curation Expert Panel (GN036, version 6.0.0) [19, 20]. The specification’s combining rules are those of Richards et al. [18] with two stated additions on the benign side: one Strong benign criterion alone meets likely benign, and one Strong plus three Supporting benign criteria meet benign. Copy number variants were assessed against the ACMG/ClinGen technical standards [26]. Only final classifications are reported; no comparison is drawn with any prior or preliminary assignment. The criteria applied to each variant, with their strength levels, are given in Table 1; the reasoning for each variant, together with the combining rule invoked, is given in Table 2.

**Table 2:** Criterion-by-criterion classification rationale for each variant (ClinGen GN036 v6.0.0)

Proband	Variant (e1)	Class	Criterion-by-criterion rationale and combining rule (ClinGen GN036 v6.0.0)
MECP2-001	p.Arg179Trp	P	Expert Panel classification adopted: pathogenic (ClinVar 143603). The Panel’s ClinVar submission cites PS2_Very Strong (at least two confirmed de novo occurrences in individuals with MECP2-related disorders), PP1_Strong (segregation across at least five informative meioses in the published literature) and PM2_Supporting; the Panel’s assessment rests in part on case-level laboratory data not available to this study. This series adds two unrelated probands (MECP2-001 and MECP2-021, no known relatedness), which are not counted towards PS4 because the variant is present in gnomAD v4.1.0 and PM2 does not apply, and PP3 (REVEL at or above 0.644). PP1 was not applied within this series: in the MECP2-021 pedigree the affected maternal uncle was not genotyped because the family declined testing, leaving a single informative meiosis, below the specification minimum of two.
MECP2-002	p.Cys425Ser	VUS	PM2_Supporting (absent from gnomAD v4.1.0) and PS4_Supporting (second independent occurrence: two unrelated probands in this series, MECP2-002 and MECP2-018). Two Supporting criteria meet no pathogenic or likely pathogenic rule. Uncertain significance.
MECP2-003	p.Pro415Argfs*3	VUS	PVS1 at Very Strong under the specification’s MECP2-specific rule: the first altered residue, e2 Pro403, lies proximal to p.Glu472. PM2_Supporting (absent from gnomAD v4.1.0). PS4 is not applicable: the only ClinVar record of the identical variant is this study’s own submission, and no independent published occurrence was identified. One Very Strong plus one Supporting criterion meets no pathogenic or likely pathogenic rule under the specification. Uncertain significance.
MECP2-004	p.Pro399Hisfs*19	P	PVS1 at Very Strong under the specification’s MECP2-specific rule: the first altered residue, e2 Pro387, and the new stop codon, e2 residue 405, both lie proximal to p.Glu472. PM2_Supporting (absent from gnomAD v4.1.0). PS4_Supporting: RettBASE (ClinVar 143373) records the identical variant in two females with Rett syndrome, one classical and recorded as de novo and one recorded as not certain, reported in two French cohort studies with overlapping authorship and patient sources [26, 27]. The two were counted as a single observation; that individual is female and demonstrably different from this male proband, so together they give a second independent occurrence. PM6 was not applied in addition, so that the same published individual is not counted under two criteria; the classification does not depend on it. BP5 was not applied: the proband’s reported CACNA1A stop-gain variant is recorded without HGVS nomenclature or segregation data and was not independently classified (Table 4), so it could not be established as an alternate molecular basis of disease. One Very Strong plus two Supporting criteria meets pathogenic, the combination on which the Expert Panel classified the C-terminal frameshift e2 p.Val380fs as pathogenic (ClinVar 143345). The reading-frame study cited in the Discussion [17] models this exact variant (CTD3) in the mouse, finds wild-type MeCP2 protein and transcript levels with no Rett-like phenotype, and predicts it to be benign; the specification restricts BS3 to RNA studies, so these data were not applied, and the conflict with the classification is set out in the Discussion. Pathogenic.
MECP2-005	p.Arg280Trp	VUS	Expert Panel classification adopted: uncertain significance (ClinVar 143700). The Panel’s submission cites BS2 (at least two unaffected individuals in published and internal laboratory data), BP5 (occurrence in a patient with an alternate molecular basis of disease) and PP3, a conflict resolved as uncertain. In gnomAD v4.1.0 the variant has 26 exome alleles with 3 hemizygotes and 4 genome alleles with 1 hemizygote; the group-maximum filtering allele frequency (1.9 per 100,000, non-Finnish European) meets the specification’s BS1 range and PP3 is also met, so our own derivation would likewise resolve as uncertain, concordant with the Panel.
MECP2-006	p.Thr255Pro	VUS	PM2_Supporting (absent from gnomAD v4.1.0) only. One Supporting criterion meets no rule. Uncertain significance.
MECP2-007	p.Pro373Arg	VUS	Present in gnomAD v4.1.0 (1 allele, 1 South Asian hemizygote), so PM2 does not apply; no phenotype information exists for that hemizygote, so BS2 is not applicable under the specification’s instruction. No criterion applied. Uncertain significance.
MECP2-008	p.Arg200Trp	B	Expert Panel classification adopted: benign (ClinVar 566728, Specifications v5.0). The Panel’s submission cites BS1 (highest sub-population minor allele frequency 1.76 per 100,000 in the South Asian population, above the threshold of 0.83 per 100,000) and BS2 (at least two unaffected individuals in internal laboratory data). On the public data available to us (8 exome alleles, 2 hemizygotes, allele number 1,098,231; 1 genome allele; group-maximum filtering allele frequency 2.6 per million), neither criterion would have been asserted under the operational rules stated in the Methods; the adopted classification rests on the Panel’s fuller data.
MECP2-009	p.Glu181Gly	VUS	Present in gnomAD v4.1.0 (1 allele, 0 hemizygotes), so PM2 does not apply. No criterion applied. Uncertain significance.
MECP2-010	p.Arg96Trp	VUS	Present in gnomAD v4.1.0 (2 alleles, 0 hemizygotes), so PM2 does not apply. PM1 is not applicable: the affected residue is e2 Arg84 (e1 Arg96), proximal to the specification’s methyl-CpG-binding domain range of e2 residues 90 to 162. No criterion applied. Uncertain significance.
MECP2-011	p.Ala152Val	LP	PS4 is not applicable: the variant is present in gnomAD v4.1.0 (2 alleles including 1 hemizygote), so PM2 does not apply, and the specification instructs that PS4 is not used where PM2 does not apply. The four probands in this series (MECP2-011, -012, -013 and − 020), from different provinces and with no known relatedness, are reported as observations only. PP1_Strong: in the published PPM-X family of Klauck et al. [12], not counting the two affected males screened first (II-6 and III-7), six informative meioses co-segregate with the variant: I-1 to the affected son II-2 (variant present) and to the unaffected son II-17 (variant absent); II-3 to the affected son III-1; II-19 to the affected son III-20 and to the unaffected son III-19 (variant absent); and the obligate carrier II-11 to the unaffected son III-8 (variant absent). In the four-generation family of Lambert et al. [15], the variant was present in all seven affected relatives tested and absent from the two unaffected relatives tested; the obligate carrier I-2 to her affected sons II-1, II-4 and II-5 and the carrier II-3 to her affected son III-5 give three further informative meioses after one of the four is set aside as the index, since the order of testing is not reported. Female offspring, who also co-segregate in this family, are not counted. The carrier females of both families are reported as mildly affected [12, 15] and do not meet the specification’s condition for BS2; unaffected heterozygous carriers were not counted towards BS2 (see Methods). Nine informative meioses across the two families meet PP1_Strong, and six on the stricter count that uses transmissions to affected males only. PM1 (e2 residue 140 within the methyl-CpG-binding domain, 90 to 162). PP3 (REVEL at or above 0.644). No phenotype information exists for the gnomAD hemizygote, so BS2 is not applicable. BS1 is not asserted: the variant is present as a single allele in each of two sub-populations (African/African American, 1 of 26,381 alleles; East Asian, 1 of 30,198), frequencies that fall numerically within the specification’s BS1 range, but a single allele does not meet the allele-count condition stated in the Methods, and the group-maximum filtering allele frequency does not reach the BS1 range. One Strong (PP1_Strong) plus one Moderate (PM1) meets likely pathogenic. The single gnomAD hemizygote is consistent with a hypomorphic allele of reduced penetrance and is not evidence of clinical normality.
MECP2-012	p.Ala152Val	LP	As for MECP2-011: the same recurrent allele, likely pathogenic on PP1_Strong, PM1 and PP3 (one Strong plus one Moderate).
MECP2-013	p.Ala152Val	LP	As for MECP2-011: likely pathogenic on PP1_Strong, PM1 and PP3. The proband’s mother is clinically affected with mild intellectual disability; molecular confirmation could not be obtained for this study. The family is consanguineous.
MECP2-014	p.Arg202Cys	VUS	Expert Panel classification adopted: uncertain significance (ClinVar 156667, Specifications v3.0). The Panel’s submission cites BS2 (at least two unaffected individuals in internal laboratory data) and PS3_Supporting (a quantitative localisation assay showing impact on protein function), a conflict resolved as uncertain, and records REVEL at 0.626, below the PP3 threshold. In gnomAD v4.1.0 the variant has 8 exome alleles with 2 hemizygotes and an allele number of 1,098,224; the group-maximum filtering allele frequency (2.6 per million) does not reach the BS1 range under the operational rule stated in the Methods.
MECP2-015	p.Lys375Glnfs*30	VUS	PVS1 at Very Strong under the specification’s MECP2-specific rule (first altered residue e2 Lys363, proximal to p.Glu472). PM2_Supporting (absent from gnomAD v4.1.0; allele number 1,097,238 at the site). PS4 was not applied at any strength. The identical variant is classified as pathogenic in ClinVar by six independent clinical diagnostic laboratories, and each submission was read individually for a documented affected individual: one records a single de novo occurrence without phenotypic description; one states explicitly that the variant has not been reported in the literature in individuals affected with MECP2-related conditions and classifies it from the pathogenicity of other variants in the same region; two argue from predicted truncation and from comparable downstream variants; and two provide no supporting narrative. The number of submitting laboratories is therefore not a count of phenotyped, unrelated affected individuals, overlap between submissions cannot be excluded, and this study’s own submission is excluded in any case. One Very Strong plus one Supporting criterion meets no pathogenic or likely pathogenic rule. Uncertain significance. The variant lies upstream of the C-terminal deletion-prone region to which the reading-frame model applies [17], and the proband has a severe presentation with a refractory Lennox-Gastaut course, which we bring forward as an explicit qualifier while noting that the variant is outside that region.
MECP2-016	c.*35_*287del	VUS	Deletion of approximately 253 base pairs lying entirely within the 3’ untranslated region (c.*35_*287del on the coordinates stated by the referring laboratory, which are treated as approximate); no coding nucleotide is removed. PVS1 and PM4 are inapplicable and no functional data exist. The gnomAD v4 structural variant callset was queried for the region: no focal overlapping deletion has been called, and mean genome coverage across the interval is approximately 24-fold; given the limited sensitivity of short-read callsets for events of this size, this absence is weak evidence at most. No pathogenic or benign criterion applies. Uncertain significance. The reported interval contains the proximal polyadenylation signal; a regulatory effect cannot be excluded but is unproven and was not used in classification.
MECP2-017	p.Met430Val	VUS	Present in gnomAD v4.1.0 (2 alleles, 1 hemizygote, no phenotype information). No criterion applied. Uncertain significance.
MECP2-018	p.Cys425Ser	VUS	PM2_Supporting (absent from gnomAD) and PS4_Supporting (second independent occurrence, with MECP2-002). Two Supporting criteria meet no rule. Uncertain significance.
MECP2-019	p.Pro445Glnfs*49	VUS	PVS1 at Very Strong under the specification’s MECP2-specific rule: the first altered residue of e2 p.Pro433Glnfs*49 is Pro433, proximal to p.Glu472; the new stop codon, at e2 residue 481, lies distal of p.Glu472 but does not determine the branch. PM2_Supporting (absent from gnomAD v4.1.0). Clinical evidence, assessed separately: BS2 at Strong, since the identical variant was detected in the proband’s two adult brothers, both hemizygous and both clinically unaffected on assessment; the proband, tested for congenital absence of teeth and with normal mental and motor development, is not counted in this evidence. The brothers do not show that the altered C-terminal segment is dispensable and were not used to modify PVS1. The pathogenic criteria meet no pathogenic or likely pathogenic rule and the benign criterion alone would meet likely benign; the two conflict, so the variant is of uncertain significance, as the Expert Panel concluded for e2 p.Pro402LeufsTer3 on PVS1 and PM2_Supporting against BS2_Supporting and BP5 (ClinVar 1700183).
MECP2-020	p.Ala152Val	LP	As for MECP2-011: the recurrent A152V allele, likely pathogenic on PP1_Strong, PM1 and PP3 (one Strong plus one Moderate).
MECP2-021	p.Arg179Trp	P	As for MECP2-001: the Expert Panel classification (pathogenic, ClinVar 143603) is adopted, with the Panel’s cited criteria quoted there. The pedigree includes an affected maternal uncle who was not genotyped because the family declined testing, so co-segregation is not demonstrated and PP1 is not applied; the pedigree pattern is consistent with X-linked inheritance. MECP2-001 and MECP2-021 are unrelated as far as is known; PS4 is not applied, as for MECP2-001.
MECP2-022	p.Pro400Serfs*3	VUS	PVS1 at Very Strong under the specification’s MECP2-specific rule (first altered residue e2 Pro388, proximal to p.Glu472) as the only criterion. The variant is present in gnomAD v4.1.0 (1 allele, 0 hemizygotes) at a low-coverage site (allele number 535,637, against approximately 1.10 million elsewhere in the exon), so PM2 does not apply. The only ClinVar record of the identical variant is this study’s own submission, so PS4 is not applicable. A single Very Strong criterion meets no rule under the specification. Uncertain significance. This proband and MECP2-004 form the adjacent-codon pair discussed in the text as an exploratory observation.

Only final classifications are reported. Where the Expert Panel classification is adopted, the criteria quoted are those cited in the Panel’s own ClinVar submission; the Panel’s assessments rest in part on internal case-level data not available to this study. For classifications derived in this study, the combining rule met is named in the row; the specification’s combining rules are those of Richards et al. [18] with the stated benign-side additions (one Strong benign criterion meets likely benign; one Strong plus three Supporting benign criteria meet benign). PVS1 strength followed the specification’s MECP2-specific rule: nonsense and frameshift variants up to p.Glu472 (e2) meet PVS1 at Very Strong and truncating variants distal of p.Glu472 meet PVS1_Moderate; the specification does not state which position applies to a frameshift, and the first altered residue, from which the normal sequence is lost, was used rather than the new stop codon; this choice determines the branch for MECP2-019 only, which is of uncertain significance on either reading. The reading-frame observation played no part in classification. PS4 is not used where PM2 does not apply or BS1 is applied, as the specification instructs. PS4 thresholds: at least five observations for Strong, three to four for Moderate, a second independent occurrence for Supporting; unrelated probands in this series and unrelated published families count once each, and this study’s own ClinVar submission is excluded from every count. External ClinVar submissions of the identical variant were counted as PS4 observations only where the submission itself documents a phenotyped, unrelated affected individual, since the number of submitting laboratories is not equivalent to the number of independent probands; submissions resting on predicted truncation, on comparison with other variants in the same region, or on an assertion without a described individual were not counted. Observations that might describe the same individual were counted once, and an external observation was counted alongside a proband of this series only where it was demonstrably a different individual, as the specification requires. PM6 was not applied to an individual already counted towards PS4. Second-locus findings reported by the referring laboratories were not used for BP5, since none could be established as an alternate molecular basis of disease. PP1 in published families: an informative meiosis is a transmission from a mother of confirmed or obligate carrier status to a genotyped son whose genotype matches his affection status; female offspring and the affected individuals screened first, or one affected male where the order of testing is not reported, are not counted; two, three to four and at least five meioses meet PP1, PP1_Moderate and PP1_Strong. PM1 ranges (e2 numbering): methyl-CpG-binding domain, residues 90 to 162; NCoR/SMRT interaction hotspot, residues 302 to 306. PP3 threshold: REVEL at or above 0.644. BS2 requires phenotyped unaffected individuals under the specification; gnomAD hemizygotes without phenotype information were never coded as BS2, and, since the ACMG/AMP definition of BS2 requires full penetrance to be expected in the individual observed [18] and the Expert Panel’s methods confine the criterion to individuals whose zygosity would normally be consistent with disease [20], unaffected heterozygous females were not counted for variants assessed as causes of disease in hemizygous males. BS1 was asserted in our own derivations only where a variant was observed in at least five alleles within a sub-population of at least 2,000 alleles and the group-maximum filtering allele frequency met the threshold

Where the Expert Panel has itself classified the identical variant in ClinVar, we adopted the Panel’s classification, state this in the corresponding rows of Tables 1 and 2, and quote the criteria cited in the Panel’s ClinVar submission. The Panel’s assessments rest in part on case-level and internal laboratory data that are not available to this study, so adoption rather than independent rederivation is the transparent course. This applied to four variants: c.535 C > T (pathogenic; ClinVar 143603), c.838 C > T (uncertain significance; 143700), c.598 C > T (benign; 566728) and c.604 C > T (uncertain significance; 156667). All other variants were classified by us under the specification.

Population data were taken from a single source, gnomAD v4.1.0 [22], queried variant by variant using GRCh38 coordinates. Allele counts, allele numbers, hemizygote counts and the group-maximum filtering allele frequency (95% confidence, FAF95) are given in Additional file 1 (Table S1a); allele and hemizygote counts are also given in Table 1. PM2_Supporting was applied only where the variant was absent from gnomAD v4.1.0; strict absence, rather than a low-frequency threshold, is the specification’s own rule (“absent, zero observations in control databases”). The specification defines BA1 and BS1 as sub-population frequency thresholds (at least 0.0083% and at least 0.00083%, respectively, in any continental sub-population of at least 2,000 alleles). Because a single allele in a sub-population of 20,000 to 60,000 alleles arithmetically exceeds the BS1 threshold, frequencies driven by one or two observations would otherwise be treated as population evidence. The Sequence Variant Interpretation Working Group asks that a variant be observed in at least five alleles within a population group of at least 2,000 alleles before BA1 or BS1 is asserted, a condition that almost all Expert Panels apply [27]; the gene-specific document restates the 2,000-allele condition but not the allele count. In our own derivations we therefore asserted BS1 only where a variant met this allele count in a qualifying sub-population and where the gnomAD group-maximum filtering allele frequency also met the threshold. Adopted Expert Panel classifications were not altered by this rule. Among the variants classified by us, none reaches five alleles in any single sub-population of gnomAD v4.1.0, so the two conditions give the same result. BS2 was applied only for individuals with documented phenotype, following the specification’s instruction that the criterion “should be applied in cases where the healthy adult is devoid of neurodevelopmental phenotypes” and is best used “with internal curated data that includes clinical information or published patients that have been phenotyped”. gnomAD hemizygotes, for whom no phenotype information exists, are therefore reported descriptively in Tables 1 and 2 but were never coded as BS2 in our own derivations. Under the specification, two phenotyped unaffected hemizygotes or heterozygotes meet BS2 at Strong and one meets BS2 at Supporting. The ACMG/AMP definition of BS2 requires that full penetrance be expected in the individual observed, in the heterozygous state for a dominant disorder and in the hemizygous state for an X-linked one [18], and the Expert Panel’s methods confine the criterion to individuals whose zygosity would normally be consistent with disease [20]. For variants assessed as causes of disease in hemizygous males, unaffected hemizygous males were therefore counted, and unaffected heterozygous females, including the carrier mothers in this series, were not. Of the variants classified by us, this rule affects the class of p.Ala152Val; for the other variants with an identified carrier mother in this series (c.1274G > C, c.286 C > T and c.1334del), counting her would leave the classification unchanged.

PM1 was applied within the methyl-CpG-binding domain (e2 residues 90 to 162) and the NCoR/SMRT interaction hotspot of the transcriptional repression domain (e2 residues 302 to 306), the residue ranges and numbering of the specification. PP3 was applied where REVEL was at or above 0.644, the specification’s calibrated threshold [28]. PS4 was applied only to variants meeting PM2_Supporting and not meeting BS1, as the specification instructs, and against its thresholds of at least five observations for PS4, three to four for PS4_Moderate and a second independent occurrence for PS4_Supporting, counting once per unrelated family and drawing on unrelated probands in this series and unrelated published families. Records arising from this study’s own ClinVar submission were excluded from every count. External ClinVar submissions of the identical variant were counted as PS4 observations only where the submission itself documents a phenotyped, unrelated affected individual. The number of submitting laboratories is not equivalent to the number of independent probands: laboratories may rely on the same published case, may submit overlapping data, and may assert a classification from sequence-level reasoning without an observed individual. Every external submission for the variants concerned was therefore read individually, and submissions resting on predicted truncation, on comparison with other variants in the same region, or on an assertion without a described individual were not counted. Published or database observations that might describe the same individual, such as reports from cohorts with overlapping authorship, were counted as a single observation. As the specification requires, an external observation was counted alongside a proband of this series only where it was demonstrably a different individual. Where no external source documented a phenotyped affected individual, only the probands of this series were counted, and a single proband does not meet PS4. PP1 requires at least two informative meioses even at Supporting strength under the specification and could not be applied in any family in this series. Co-segregation in published families was assessed where the report gives the genotypes of affected and unaffected relatives. An informative meiosis was counted as a transmission from a mother of confirmed or obligate carrier status to a genotyped son whose genotype matched his affection status. Female offspring were not counted, since the phenotype of heterozygous females is mild and variable, and the affected individuals screened first in the family, or one affected male where the report does not give the order of testing, were not counted. The specification’s thresholds of two, three to four and at least five informative meioses were applied for PP1, PP1_Moderate and PP1_Strong. For predicted loss-of-function variants, PVS1 strength followed the specification’s MECP2-specific rule [19, 20], which takes precedence over the general decision tree of Abou Tayoun et al. [21]: nonsense and frameshift variants up to p.Glu472 (e2) meet PVS1 at Very Strong, truncating variants distal of p.Glu472 meet PVS1_Moderate, and a frameshift that reads through the stop codon meets PVS1. The specification does not state which position applies to a frameshift; we used the first altered residue, from which the normal sequence is lost, and not the new stop codon. This choice determines the branch for one variant only (MECP2-019), and that variant is of uncertain significance on either reading, since PVS1_Moderate with PM2_Supporting would equally conflict with BS2 at Strong. PVS1 strength was not modulated by reading frame. The functional study discussed in the Discussion [17] was not applied as a criterion, since the specification restricts BS3 to RNA studies of splicing and transcript composition. Second-locus findings reported by the referring laboratories were not used for BP5, since none could be established as an alternate molecular basis of disease (see Phenotypic data and handling of missing data, and Table 4). Terminology follows the ClinGen Variant Classification Working Group, which has assumed the work of the former Sequence Variant Interpretation Working Group.

The 17 distinct variants identified in the 22 probands have been submitted to ClinVar by this study (Submission ID SUB16291306), together with the criteria applied and their strength levels; the ClinVar records will be updated to match the final classifications reported here.

### Statistical analysis

The analysis is descriptive throughout. No formal statistical comparisons were performed, no missing data were imputed, and every proportion is reported as n/N, where N is the number of probands with a recorded value for that variable; denominators therefore differ between variables.

### Cohort structure

Two levels are distinguished throughout. The ascertained series comprises all 22 probands found to carry a MECP2 variant, and all are reported and deposited. The analysis cohort comprises the 21 probands whose variants were classified as pathogenic, likely pathogenic or of uncertain significance. All phenotypic proportions and all statements about the clinical range in this paper rest on the analysis cohort. The proband whose variant was classified as benign (MECP2-008) is reported in a separate section and contributes to no phenotypic denominator. Within the analysis cohort, phenotypic findings are presented separately for probands with pathogenic or likely pathogenic variants (*n* = 7) and for probands with variants of uncertain significance (*n* = 14).

### Phenotypic data and handling of missing data

Phenotypic data were extracted retrospectively from diagnostic records. No prospective or uniform phenotyping protocol was applied, and the depth of recording varied between centres and between probands. Missing values were not imputed. Fields without a record are marked as not recorded and are excluded from the corresponding denominator, so that denominators differ between headings. Every proportion in the Results is therefore given as n/N with the denominator identified. Ages are reported as recorded: the age at the most recent documented assessment could be retrieved in years for seven probands; for the remainder, only the age bracket at testing (child, adolescent or adult) was recorded in the assembled records, and exact ages could not be retrieved retrospectively. Growth and head-circumference findings are reported as recorded by the referring clinicians in the source documents; the underlying measurements, standard deviation scores and growth references could not be retrieved retrospectively, no uniform definition or cut-off could therefore be applied, and these findings are treated as clinician-recorded observations rather than as standardised anthropometry (see Limitations). Secondary and incidental findings are reported as recorded in the referring laboratories’ diagnostic reports and are given in Table 4, which states for each the nomenclature available, the classification as reported, whether segregation data exist, and whether the finding arose incidentally during diagnostic analysis or was sought actively. Complete variant nomenclature with transcript is recorded in the source documents for one of the three findings only; for the other two the reports give the gene and the variant type alone. No ACMG/AMP class is stated in the retrievable report for two of the three, and no formal segregation study was available for any of them. These findings were therefore not reclassified for this study under the criteria applied to the MECP2 variants; they are reported as documented, and no independent confirmation of their classification is claimed (see Limitations).

## Results

### Variant spectrum

The 22 probands of the ascertained series carried 17 distinct MECP2 variants: 11 missense variants, 5 frameshift variants and 1 deletion confined to the 3’ untranslated region. Sixteen probands carried missense variants, 5 carried frameshift variants and 1 carried the 3’ untranslated region deletion. Three variants were recurrent: p.Ala152Val (e1) in four unrelated probands (MECP2-011, -012, -013 and − 020), p.Arg179Trp in two (MECP2-001 and − 021) and p.Cys425Ser in two (MECP2-002 and − 018). All variants and their e2 (MANE Plus Clinical) equivalents, domains, gnomAD v4.1.0 frequencies, applied criteria and final classifications are given in Table 1, and the reasoning for each is given in Table 2. The spectrum across the whole protein is shown in Fig. 2.

The five frameshift variants all lie within the large final exon of MeCP2_e1, which encodes the C-terminal domain, and not within the penultimate exon. Two pairs converge on identical downstream stop codons: MECP2-003 (c.1243_1252del) and MECP2-004 (c.1194_1203del) terminate at the same out-of-frame stop, as do MECP2-015 (c.1122dup) and MECP2-022 (c.1198_1202del). The region contains short repeat sequences that favour misalignment by replication slippage.

The deletion in MECP2-016 is reported by the referring laboratory at chrX:154,030,080–154,030,332 (GRCh38), spanning 253 base pairs; as set out in the Methods, these breakpoints are treated as approximate. On the reported coordinates, the MeCP2_e1 coding sequence ends at chrX:154,030,367, so the deletion begins 35 base pairs downstream of the stop codon and lies entirely within the 3’ untranslated region; it is designated NM_001110792.2:c.*35_*287del. No coding nucleotide is removed. The reported interval contains the annotated 3’ end of the short-3’UTR MECP2 transcript and two canonical AATAAA hexamers, at chrX:154,030,259 − 154,030,264 and chrX:154,030,325 − 154,030,330. We queried the gnomAD v4 structural variant callset for this region: no focal deletion overlapping the interval has been called, the only overlapping calls being megabase-scale rearrangements, and mean gnomAD v4 genome coverage across the interval is approximately 24-fold, with partial exome coverage as expected for an untranslated region. Short-read structural variant callsets have limited sensitivity for events of this size, so this absence is weak evidence at most.

### Classification

Under the GN036 v6.0.0 specification, of the 17 distinct variants 2 were classified as pathogenic, 1 as likely pathogenic, 13 as of uncertain significance and 1 as benign. Because three variants recur, the corresponding proband-level counts across the 22 probands of the ascertained series are 3, 4, 14 and 1. For the four variants that the Expert Panel has itself classified in ClinVar, the Panel’s classification was adopted, as set out in the Methods: c.535 C > T as pathogenic (ClinVar 143603), c.838 C > T and c.604 C > T as of uncertain significance (143700 and 156667) and c.598 C > T as benign (566728). The criteria cited in the Panel’s own ClinVar submissions are reproduced in Table 2, together with the observations this series adds for each of these variants. All other classifications were derived by us under the specification, and Table 2 names the combining rule met in each case.

p.Ala152Val is present in gnomAD v4.1.0 with an allele count of 2 including 1 hemizygote, so PM2_Supporting does not apply, and under the specification’s instruction PS4 is therefore not applicable. The four probands in this series who carry the variant, drawn from different provinces and with no known relatedness, are reported as observations and are not counted towards any criterion. PM1 applies, since e2 residue 140 lies within the methyl-CpG-binding domain, and PP3 applies. PP1 was assessed in the published PPM-X family of Klauck et al. [12], in which the variant was present in all genotyped affected males and absent from the genotyped unaffected males. Not counting the two affected males screened first (II-6 and III-7), the pedigree provides six informative meioses: the founder carrier I-1 transmitted the variant to the affected son II-2 and did not transmit it to the unaffected son II-17; the carrier II-3 transmitted it to the affected son III-1; the carrier II-19 transmitted it to the affected son III-20 and did not transmit it to the unaffected son III-19; and the obligate carrier II-11 did not transmit it to the unaffected son III-8. This family alone meets PP1_Strong. The four-generation family of Lambert et al. [15] gives consistent evidence: the variant was found in all seven affected relatives tested and was absent from the two unaffected relatives tested. The obligate carrier I-2 transmitted it to her three affected sons II-1, II-4 and II-5, and the carrier II-3 to her affected son III-5; with one of these four treated as the index, since the order of testing is not reported, the family adds three informative meioses. Across the two families there are nine informative meioses. Counting only the transmissions to affected males and setting aside those to unaffected sons, the two families still give six, so the threshold of five is met on either count. BS1 is not asserted: the variant is present as a single allele in each of two sub-populations (African/African American, 1 of 26,381 alleles; East Asian, 1 of 30,198), frequencies that fall numerically within the specification’s BS1 range but do not meet the allele-count condition stated in the Methods. The carrier females of both families are reported as mildly affected [12, 15], so they do not meet the specification’s condition for BS2, and unaffected heterozygous carriers were not counted towards BS2, as set out in the Methods. One Strong criterion (PP1_Strong) plus one Moderate criterion (PM1) meets likely pathogenic, and PP3 is additional.

Among the frameshift variants, PVS1 strength was assigned by the specification’s MECP2-specific rule. In MECP2-003, -004, -015 and − 022 the first altered residue is e2 Pro403, Pro387, Lys363 and Pro388 respectively, all proximal to p.Glu472, as are their new stop codons, so all four meet PVS1 at Very Strong. For p.Pro399Hisfs*19 (MECP2-004) the identical variant has been observed in females with Rett syndrome. RettBASE (ClinVar 143373) records two observations, one described as classical and recorded as de novo and one recorded as not certain, which trace to two French cohort reports [23, 24]. These reports share authors and patient sources, so the two observations may describe a single individual, and they were counted as one. That individual is female and the proband of this series is male, so the two are demonstrably different individuals, as the specification requires, and together they give a second independent occurrence: PS4_Supporting. The variant is absent from gnomAD v4.1.0, and PM2_Supporting applies. PVS1 with two Supporting criteria meets pathogenic under the specification’s combining rules; the Expert Panel classified the C-terminal frameshift e2 p.Val380fs as pathogenic on the same combination of PVS1, PS4_Supporting and PM2_Supporting (ClinVar 143345). PM6 was not applied in addition, so that the same published individual is not counted under two criteria; the classification does not depend on it. BP5 was not applied, since the proband’s reported CACNA1A finding could not be established as an alternate molecular basis of disease (Table 4). The reading-frame study discussed below models this variant in the mouse and predicts it to be benign [17]; because the specification restricts BS3 to RNA studies, these data were not applied, and their bearing is considered in the Discussion. For p.Lys375Glnfs*30 (MECP2-015) the identical variant is classified as pathogenic in ClinVar by six independent clinical diagnostic laboratories, and each submission was read individually for a documented affected individual. One laboratory records a single de novo occurrence without any phenotypic description; one states explicitly that the variant has not been reported in the literature in individuals affected with MECP2-related conditions and classifies it from the pathogenicity of other variants in the same region; two argue from predicted truncation and from comparable downstream variants; and two provide no supporting narrative. No submission establishes a phenotyped, unrelated affected individual, so only the proband of this series can be counted, and PS4 is not met. For p.Pro415Argfs*3 (MECP2-003) no external record exists at all beyond this study’s own submission. In MECP2-003 and − 015, PVS1 with PM2_Supporting meets no pathogenic or likely pathogenic rule under the specification’s combining rules. In MECP2-022, where PM2 does not apply because the variant is present in gnomAD v4.1.0, PVS1 stands alone and likewise meets no rule. These three variants are of uncertain significance. The Expert Panel has classified a C-terminal frameshift on the same combination, e2 p.His368LeufsTer18 with PVS1 and PM2_Supporting, as of uncertain significance (ClinVar 3775030). For MECP2-016, no pathogenic or benign criterion was applicable; the coding sequence is unaffected, and functional data are absent.

MECP2-019 carried c.1334del, p.Pro445Glnfs*49 (e2 p.Pro433Glnfs*49). The first altered residue, e2 Pro433, lies proximal to p.Glu472, whereas the new stop codon, at e2 residue 481, lies distal of it; the rule is applied to the first altered residue, as set out in the Methods, so the variant meets PVS1 at Very Strong. The variant is absent from gnomAD v4.1.0, and PM2_Supporting applies. The clinical evidence was assessed separately. The proband was tested for congenital absence of teeth, the variant was identified in the analysis because of its rarity, and his mental and motor development were recorded as normal. The variant was subsequently detected in his two adult brothers, both hemizygous and both clinically unaffected on assessment; the proband himself is not counted in this evidence. Two phenotyped unaffected hemizygotes meet BS2 at Strong. The brothers do not demonstrate that the altered C-terminal segment is functionally dispensable, and they were not used to modify PVS1. The pathogenic criteria meet no pathogenic or likely pathogenic rule, the benign criterion alone would meet likely benign, and the two sets of evidence conflict, so the variant is of uncertain significance. The Expert Panel resolved a comparable conflict in the same way for e2 p.Pro402LeufsTer3, where PVS1 and PM2_Supporting stood against BS2_Supporting and BP5 (ClinVar 1700183). We report the variant, and have deposited it in ClinVar, so that other groups encountering it have access to the evidence.

### Clinical findings in probands with pathogenic or likely pathogenic variants (*n* = 7)

The seven probands were MECP2-001, -004, -011, -012, -013, -020 and − 021. Exact ages were recorded for six (3 years 11 months to 11 years 9 months); the remaining proband (MECP2-021) was a child of unrecorded exact age at the last documented assessment. Intellectual disability or developmental delay was present in 7 of 7, and severity was graded in all seven: severe in 1 of 7 (MECP2-021), moderate in 2 of 7 (MECP2-001 and − 011), mild in 3 of 7 (MECP2-004, -012 and − 013) and mild to moderate in 1 of 7 (MECP2-020). Four of the seven carry the same recurrent allele, p.Ala152Val.

Seizure data were available for only 3 of 7 probands. Two had seizures, with onset at approximately 1 year (MECP2-013) and 2 years (MECP2-001), and one (MECP2-004) had no seizures and a normal electroencephalogram. With a denominator of three, this proportion carries no weight and is reported only for completeness. Brain magnetic resonance imaging findings were recorded for one proband in this group, showing volume loss with mega cisterna magna (MECP2-001); per-proband neuroimaging availability could not be reconstructed from the assembled records, so no denominator is offered for imaging.

Autism spectrum disorder was diagnosed in 4 of 7 probands (MECP2-004, -011, -013 and − 021). Other neuropsychiatric features recorded in this group were attention-deficit/hyperactivity disorder in 2 of 7 (MECP2-004 and − 011), obsessive-compulsive disorder in 1 of 7 (MECP2-001), aggression in 2 of 7 (MECP2-001 and − 013), self-directed harm in 1 of 7 (MECP2-013) and hyperactivity with a happy demeanour in MECP2-012. Growth or head-circumference findings were recorded for 4 of 7 probands, and obesity or overgrowth was present in all four: morbid obesity with severe short stature (MECP2-001), obesity with borderline microcephaly and birth macrosomia of 5000 g (MECP2-011), paediatric morbid obesity with early-onset dysglycaemia (MECP2-012) and tall stature with gynaecomastia and scoliosis (MECP2-020). Microcephaly was recorded in 1 of 4, borderline (MECP2-011), and short stature in 1 of 4 (MECP2-001).

### Clinical findings in probands with variants of uncertain significance (*n* = 14)

The fourteen probands were MECP2-002, -003, -005, -006, -007, -009, -010, -014, -015, -016, -017, -018, -019 and − 022. An exact age was recorded for one, 47 years (MECP2-022); the others were children, with one adolescent (MECP2-016) and one adult university student (MECP2-019), at the last documented assessment, and their exact ages could not be retrieved. Cognitive status was recorded for 10 of 14. Intellectual disability or developmental delay was present in 8 of these 10: severe in MECP2-003, moderate in MECP2-002, -017 and − 018, developmental delay in MECP2-014, developmental delay with intellectual disability of unstated grade in MECP2-015, and mild in MECP2-010 and − 016. MECP2-022 had no childhood intellectual disability, and MECP2-019 had normal mental and motor development.

Seizure data were available for 10 of 14 probands, and seizures were present in 8 of these 10. Age at onset ranged from infancy (MECP2-015) and 6 months (MECP2-014) to 7 years (MECP2-006), with onset at 9 months in MECP2-009, 1.5 years in MECP2-005 and 5 years in MECP2-007. MECP2-015 had a refractory Lennox-Gastaut course requiring multiple antiseizure medications and adrenocorticotropic hormone. MECP2-016 had focal dyscognitive seizures treated with three antiseizure medications. MECP2-022 had late-onset status epilepticus. MECP2-002 and MECP2-017 had no seizures; MECP2-017 had subclinical epileptiform discharges. Brain magnetic resonance imaging findings were recorded for three probands in this group, a retrocerebellar mega cisterna magna (MECP2-002), a suspected cortical malformation (MECP2-009), and white matter lesions with cerebellar vermis hypogenesis and a retrocerebellar cyst (MECP2-015); as above, no imaging denominator is offered.

Autism spectrum disorder was diagnosed in 2 of 14 probands (MECP2-017 and − 018), with autistic features noted but not formally diagnosed in MECP2-009. MECP2-022 developed late-onset psychosis. Growth or head-circumference findings were recorded for 5 of 14 probands. Microcephaly was recorded in 3 of these 5: severe at minus 4 standard deviations with low weight and persistent vomiting in MECP2-002, without further qualification in MECP2-003, and with brachycephaly in MECP2-010. Obesity was recorded in 1 of 5 (MECP2-010, in whom the source document records a body mass index of 30.5; a standard deviation score could not be retrieved). Short stature was recorded in 2 of 5 (MECP2-006 and MECP2-015).

MECP2-022 was the oldest proband, a man of 47 years who died in 2025. He had no childhood intellectual disability and developed adult-onset parkinsonism, late-onset psychosis and status epilepticus. MECP2-010 was born at 25 weeks of gestation with hypoxic-ischaemic encephalopathy.

### Growth and head circumference across the analysis cohort

Growth or head-circumference findings were recorded for 9 of the 21 probands in the analysis cohort. Among these nine, obesity or overgrowth was recorded in 5, microcephaly in 4 (one of them borderline) and short stature in 3. Because MECP2-010 was born at 25 weeks of gestation with hypoxic-ischaemic encephalopathy, a sufficient explanation for his phenotype on its own, the same counts excluding this proband are 4 with obesity or overgrowth, 3 with microcephaly and 3 with short stature among 8 probands with recorded findings. These are counts of clinician-recorded observations without uniform definitions or reference charts, as set out in the Methods, and no comparative claim is attached to them.

### Inheritance and segregation

A maternal carrier was identified in 6 of the 22 families of the ascertained series (MECP2-001, -002, -010, -019, -020, -021), and in each of these the variant was maternally inherited. In five families the carrier state was confirmed by laboratory testing available to this study; in the sixth (MECP2-021), it rests on the referring clinician’s documented statement, the laboratory record itself not being retrievable. The mothers of the remaining 16 probands were not tested, so no de novo event was confirmed and these data do not permit an estimate of the proportion of de novo events in males. Table 3 records, for every proband, whether the mother was tested, together with the outcome. The mother of MECP2-013 is clinically affected, with mild intellectual disability, but molecular confirmation could not be obtained for this study, so she is not counted among the identified maternal carriers; this family is consanguineous. The family of MECP2-021 includes an affected maternal uncle, a history consistent with X-linked inheritance; the uncle was not genotyped because the family declined testing, so co-segregation was not demonstrated and PP1 was not applied. MECP2-001 and MECP2-021, who carry the same variant, are unrelated as far as is known; because the variant is present in gnomAD v4.1.0, PS4 was not applied to it in this series. Paternal history was recorded in some families, including a history of febrile convulsion in the father of MECP2-005; such history is not informative for the inheritance of an X-linked locus. Maternal testing status, outcomes and findings in carrier mothers are given in Table 3.

**Table 3:** Per-proband phenotypic features, test indication and maternal testing (analysis cohort, *n* = 21)

Proband	Age	Class	Cognition	Seizures (onset)	Neuropsychiatric	Growth / OFC	Neuroimaging	Test indication	Maternal testing	Inheritance / family notes
MECP2-001	6 y	P	Moderate ID	Yes (~ 2 y)	OCD; aggression	Morbid obesity; severe short stature	Volume loss; mega cisterna magna	DD with dysmorphism	Tested; carrier	Maternally inherited
MECP2-004	3 y 11 mo	P	Mild ID	No (normal EEG)	ASD; ADHD	NR	NR	DD / ASD	Not tested	
MECP2-011	10 y 9 mo	LP	Moderate ID	NR	ASD (5 y); ADHD (6 y)	Obesity; borderline microcephaly; birth 5000 g	NR	Unexplained ID with neuropsychiatric features / ASD	Not tested	
MECP2-012	4 y	LP	Mild ID	EEG not done	Hyperactivity; happy demeanour	Normal OFC; paediatric morbid obesity; dysglycaemia	NR	DD	Not tested	
MECP2-013	11 y 9 mo	LP	Mild ID	Yes (~ 1 y)	Autism; aggression; self-harm	NR	NR	Unexplained ID with neuropsychiatric features	Not testable for this study	Mother clinically affected (mild ID), untested; consanguineous family
MECP2-020	11 y	LP	Mild-moderate ID	NR	NR	Tall stature; gynaecomastia; scoliosis	NR	Intellectual disability; family history	Tested; carrier	Maternally inherited
MECP2-021	Child	P	Severe ID	NR	ASD	NR	NR	Family history	Carrier per clinician report (laboratory record not retrievable)	Affected maternal uncle, not genotyped (family declined testing); pattern consistent with X-linked inheritance
MECP2-002	Child	VUS	Moderate ID	No	NR	Severe microcephaly (-4 SD); low weight; vomiting	Retrocerebellar mega cisterna magna	Unexplained ID	Tested; carrier	Maternally inherited
MECP2-003	Child	VUS	Severe ID	NR	NR	Microcephaly	NR	Unexplained ID	Not tested	Consanguineous family
MECP2-005	Child	VUS	NR	Yes (1.5 y)	NR	NR	NR	Unexplained epilepsy	Not tested	Father with febrile convulsion (not informative for X-linked inheritance)
MECP2-006	Child	VUS	NR	Yes (7 y)	NR	Short stature	NR	Unexplained epilepsy	Not tested	
MECP2-007	Child	VUS	NR	Yes (5 y)	NR	NR	NR	DD	Not tested	
MECP2-009	Child	VUS	NR	Yes (9 mo)	Autistic features (not formally diagnosed)	NR	Suspected cortical malformation	DD	Not tested	
MECP2-010	Child	VUS	Mild ID	NR	NR	Obesity (recorded BMI 30.5; SDS not retrievable); microcephaly; brachycephaly	NR	Neurodevelopmental impairment not explained by prematurity alone (born 25 wk; HIE)	Tested; carrier	Maternally inherited
MECP2-014	Child	VUS	DD	Yes (6 mo)	NR	NR	NR	Unexplained epilepsy	Not tested	
MECP2-015	Child	VUS	DD with ID	Infancy (Lennox-Gastaut; multiple ASM + ACTH)	NR	Short stature	White matter lesions; vermis hypogenesis; retrocerebellar cyst	Refractory epilepsy	Not tested	
MECP2-016	Adolescent	VUS	Mild ID	Focal dyscognitive (3 ASM)	NR	NR	NR	Refractory epilepsy	Not tested	
MECP2-017	Child	VUS	Moderate ID	No (subclinical epileptiform on EEG)	ASD	NR	NR	DD / ASD	Not tested	
MECP2-018	Child	VUS	Moderate ID	NR	ASD	NR	NR	DD / ASD	Not tested	
MECP2-019	Adult	VUS	Normal (university student)	NR	NR	NR	NR	Congenital absence of teeth	Tested; carrier	Maternally inherited; two adult brothers hemizygous for the variant and clinically unaffected
MECP2-022	47 y (died 2025)	VUS	No childhood ID	Late-onset status epilepticus	Adult-onset parkinsonism; late-onset psychosis	NR	NR	Adult-onset unexplained neurological decline	Not tested	

Probands are ordered by class, pathogenic and likely pathogenic first, then uncertain. MECP2-008 (benign) is not included and appears only in the section on the benign variant. Exact ages at the most recent documented assessment could be retrieved for seven probands, six in the pathogenic and likely pathogenic group and one in the uncertain group; for the remainder, only the age bracket at testing was recorded (see Methods), and one entry gives the age at seizure onset within the seizure column. A maternal carrier was identified in 6 of the 22 families of the ascertained series (MECP2-001, -002, -010, -019, -020, -021); five are laboratory-confirmed and one (MECP2-021) rests on the referring clinician’s documentation. ‘Maternally inherited’ is stated only for probands whose mother is an identified carrier. Denominators differ between columns because phenotyping was not uniform; NR, not recorded. OFC, occipitofrontal circumference; ID, intellectual disability; DD, developmental delay; ASD, autism spectrum disorder; ADHD, attention-deficit/hyperactivity disorder; OCD, obsessive-compulsive disorder; ASM, antiseizure medication; ACTH, adrenocorticotropic hormone; SD, standard deviation; SDS, standard deviation score; BMI, body mass index; EEG, electroencephalography; HIE, hypoxic-ischaemic encephalopathy

### Secondary findings

A second candidate locus was reported by the referring laboratory in 3 of the 22 probands of the ascertained series, all three in the analysis cohort. Two (MECP2-003 and − 019) are in the uncertain group and one (MECP2-004) is in the pathogenic and likely pathogenic group. MECP2-003, from a consanguineous family, was reported to carry a homozygous PGM3 missense variant, associated with immunodeficiency-23 and PGM3-related congenital disorder of glycosylation. MECP2-004 was reported to carry a heterozygous CACNA1A stop-gain variant, classified as likely pathogenic by the referring laboratory, and separately an in-frame CACNA1A duplication of uncertain significance. MECP2-019, whose MECP2 variant is of uncertain significance, was reported to carry a hemizygous IGSF1 nonsense variant, NM_001555.5:c.1832G > A, p.(Trp611Ter), associated with IGSF1 deficiency and central hypothyroidism. All three findings arose incidentally during diagnostic analysis and were not sought actively. IGSF1 is not on the ACMG secondary findings list, version 3.2 [29], and that finding is therefore an incidental finding rather than a secondary finding within that framework. Complete variant nomenclature with transcript is recorded in the source documents for the IGSF1 finding only; for the PGM3 and CACNA1A findings the reports give the gene and the variant type alone. No ACMG/AMP class is stated for the PGM3 and IGSF1 findings, the classification of the two CACNA1A findings is that asserted by the referring laboratory, and no formal segregation study was available for any of the three. They were therefore not reclassified for this study, and we report them as documented rather than as independently confirmed clinically relevant secondary findings. What is recorded for each is given in Table 4.

**Table 4:** Second-locus findings reported by the referring laboratories

Proband	Second locus: gene and variant nomenclature as recorded	Zygosity / inheritance	Associated condition	Classification as reported	Segregation evidence available	Incidental or actively sought
MECP2-003	PGM3, missense variant. Complete HGVS nomenclature and transcript not recorded in the retrievable report	Homozygous; consanguineous family	Immunodeficiency-23; PGM3-CDG	Reported as disease-relevant by the referring laboratory; no ACMG/AMP class stated in the retrievable report; not reclassified for this study	None. Homozygosity is consistent with the consanguinity of the family, but parental genotypes were not available	Incidental
MECP2-004	CACNA1A, stop-gain variant; separately an in-frame CACNA1A duplication. Complete HGVS nomenclature and transcript not recorded in the retrievable report	Heterozygous	Episodic ataxia / epileptic encephalopathy spectrum	Stop-gain reported as likely pathogenic and duplication as of uncertain significance by the referring laboratory; not reclassified for this study	None available	Incidental
MECP2-019	IGSF1, nonsense variant. NM_001555.5:c.1832G > A, p.(Trp611Ter), as recorded in the referring laboratory’s report	Hemizygous	IGSF1 deficiency; central hypothyroidism	Reported as disease-relevant by the referring laboratory; no ACMG/AMP class stated in the retrievable report; not reclassified for this study	None available	Incidental (not on ACMG SF v3.2 list)

All three findings arose incidentally during diagnostic analysis and were not actively sought, and each is reported here as recorded in the referring laboratory’s diagnostic report. Complete variant nomenclature with transcript is recorded in the retrievable report for the IGSF1 finding only; for the PGM3 and CACNA1A findings the reports give the gene and the variant type alone. No ACMG/AMP class is stated for the PGM3 and IGSF1 findings, and no formal segregation study was available for any of the three, so none was reclassified for this study under the criteria applied to the MECP2 variants and none should be read as an independently confirmed clinically relevant secondary finding. IGSF1 is not on the ACMG secondary findings list, version 3.2, so the IGSF1 finding is an incidental finding rather than a secondary finding within that framework. MECP2-019, in whom the IGSF1 variant was found, carries a MECP2 variant of uncertain significance. Of the three probands with a reported second candidate locus, MECP2-003 and − 019 are in the uncertain group (*n* = 14) and MECP2-004 is in the pathogenic and likely pathogenic group (*n* = 7)

### Variant classified as benign

One variant in the ascertained series was classified as benign, and the corresponding proband is excluded from all phenotypic denominators above.

MECP2-008 carried c.598 C > T, p.Arg200Trp. The Expert Panel has classified this variant as benign (ClinVar 566728), citing BS1, on a highest sub-population minor allele frequency of 1.76 per 100,000 in the South Asian population of gnomAD v4.1, and BS2, on at least two unaffected individuals in internal laboratory data; we adopted that classification. In gnomAD v4.1.0 the variant has an exome allele count of 8 with 2 hemizygotes and an allele number of 1,098,231, and one further genome allele; the group-maximum filtering allele frequency (2.6 per million) does not itself reach the BS1 threshold under the operational rule stated in the Methods, and no phenotype information exists for the gnomAD hemizygotes, so on the public data alone no criterion would have been asserted by us. The adopted Expert Panel classification rests on the Panel’s fuller data.

## Discussion

This series was ascertained through routine diagnostic testing rather than through registries of severely affected patients, and the phenotypes observed among the males it captures are graded rather than uniformly severe. Within the analysis cohort, intellectual disability ranged from mild to severe, and presentations included syndromic intellectual disability, epilepsy-predominant courses and autism-spectrum presentations. The oldest proband had an adult-onset parkinsonian course with psychosis and no childhood intellectual disability, which resembles the PPM-X end of the reported range [12, 13]; his variant is of uncertain significance, however, and the evidence available does not establish that it caused these findings, so we present the observation as a phenotype seen in a male carrying a MECP2 variant rather than as part of the MECP2-related disease spectrum. The graded picture among the probands with pathogenic or likely pathogenic variants is consistent with the progressive revision of the view that hemizygous loss of MECP2 in males is uniformly severe [8–10, 30].

We would emphasise how narrow the evidential base for this breadth is. Fourteen of the 21 probands in the analysis cohort carry variants of uncertain significance, so the claim that the phenotype is broad rests on the seven probands with pathogenic or likely pathogenic variants, four of whom carry the same recurrent hypomorphic allele. Comparison between the two groups is not possible on these data. Seizure status, for example, was recorded for only 3 of the 7 probands in the pathogenic and likely pathogenic group, against 10 of the 14 probands with uncertain variants, so the two proportions rest on denominators that are neither comparable nor large enough to interpret. The composition of each group is itself shaped by ascertainment, since testing indications in this series included refractory or unexplained epilepsy, and we therefore report the counts for each group separately and draw no comparison between them (Table 5).

**Table 5:** Published hemizygous-male MECP2 series compared with the present cohort

Study (year; journal)	Males, *n*	Age range	Ascertainment	Inheritance of informative variants	Phenotype / variant emphasis	Relevance to the present cohort
Neul et al., 2019; Am. J. Med. Genet. B [11]	30	Mostly paediatric	RTT US Natural History Study databases (registry)	Severe, frequently de novo end over-represented	Severe neonatal encephalopathy to cognitive impairment; 9 deceased (mean 9.0 y); 12/28 with neonatal or progressive encephalopathy	Registry ascertainment anchors the severe end of the male spectrum
Inuzuka et al., 2021; Eur. J. Paediatr. Neurol. [8]	98 (pool) + 8 new	4–19 y (own 8 cases)	Systematic literature review plus own clinical cohort	Severe-encephalopathy alleles de novo; ID alleles de novo or maternally inherited	Defines the three male phenotype groups (severe neonatal encephalopathy; X-linked ID-13; PPM-X)	Largest pooled male dataset and the framework this study builds on
Ananth et al., 2024; Pediatr. Neurol. [29]	59 (85 with NHS)	2–28 y; median 7 y	Parent Group for Males (advocacy)	46 (78%) de novo; 9 (15%) maternal; 4 unknown; 11 (19%) mosaic	Broad male phenotype; 27 deceased in the combined 85-male group	Advocacy ascertainment is strongly de novo-predominant; ascertainment differences preclude direct comparison of inheritance patterns
Lötjönen et al., 2025; Mol. Genet. Genomic Med. [10]	2 new + 24 reviewed	Adults (P1 23 y) plus variant-specific literature	Diagnostic case report plus A140V variant-specific review	Maternally inherited p.Ala140Val in two brothers	Wide intra- and inter-individual variability of the recurrent hypomorphic A140V/A152V allele	Directly relevant to the four A152V probands here; not used in the classification of that allele, for which PS4 is not applicable under the specification
This study, 2026	22	Children and adolescents; two adults, one of 47 y (exact ages retrievable for 7)	Multicentre diagnostic ascertainment (Türkiye)	Maternal carrier identified in 6 of 22 families (5 laboratory-confirmed, 1 clinician-documented); no confirmed de novo; maternal genotype unavailable in 16	Missense-predominant (16/22 probands); graded severity; growth or head-circumference findings recorded for 9 of 21, with obesity or overgrowth in 5 and microcephaly in 4	Diagnostic ascertainment identified milder, asymptomatic and adult-onset presentations among males carrying MECP2 variants; most of these probands carry variants of uncertain significance, so their relation to MECP2 is not established and the comparison with registry and advocacy series is qualitative

The series differ substantially in ascertainment (registry, advocacy-group, systematic-review and diagnostic); the table is a qualitative landscape rather than a basis for formal frequency comparison, and side-by-side feature rates are deliberately not tabulated. Narrative reviews are not listed as primary series. Reference numbers in brackets follow the reference list of the main text. n, number of hemizygous males; NHS, Natural History Study; ID, intellectual disability; PPM-X, pyramidal signs, parkinsonism and macroorchidism; RTT, Rett syndrome

The relationship between growth and MECP2 in males deserves attention, within the limits of what this series can show. Classic teaching emphasises microcephaly and growth restriction. In this series, growth or head-circumference findings were recorded for only 9 of the 21 probands, four of them in the pathogenic and likely pathogenic group; among the nine, obesity or overgrowth was recorded in 5 and microcephaly in 4, one of the latter borderline, and one proband had birth macrosomia of 5000 g while another had early-onset dysglycaemia. These are clinician-recorded observations from a small and unevenly phenotyped series, without uniform definitions or reference charts, and they support no comparative statement about relative frequency. They are nevertheless consistent with a growing literature on the role of MECP2 in systemic and brain metabolism [31, 32], and they suggest that growth and metabolic parameters merit attention alongside head circumference in these boys.

A second candidate locus reported in 3 of 22 probands reflects the increasing recognition that neurodevelopmental disorders are often influenced by more than one genetic factor [33]. The reported homozygous PGM3 variant in MECP2-003 and CACNA1A stop-gain in MECP2-004 may contribute to the phenotypes in those probands, and their presence means that the MECP2 variant cannot be assumed to account for the whole picture; because these findings could not be independently reclassified for this study, they are treated as reported rather than as established. After reclassification MECP2-003 falls in the uncertain group and MECP2-004 in the pathogenic and likely pathogenic group, so one of the seven probands on which the phenotypic argument principally rests carries a second candidate locus, and his mild intellectual disability, autism and attention-deficit/hyperactivity disorder may not be attributable to MECP2 alone. The same reasoning applies to paternal history. A history of an overlapping phenotype in the father is not informative for the inheritance of an X-linked locus, but it may point to undetected additional genetic susceptibility contributing to the proband’s presentation.

### Asymptomatic and mildly affected carrier mothers

In every family in which a maternal genotype was identified, the carrier mother was either clinically unaffected or, in one family, mildly affected, and this deserves comment in a gene in which heterozygous loss of function classically causes Rett syndrome. Two mechanisms, long recognised, are sufficient to account for it. First, X-chromosome inactivation: carrier females with favourably skewed inactivation of the mutant allele can be clinically normal, as documented in the earliest familial MECP2 reports, in which unaffected carrier mothers with skewed inactivation transmitted variants to affected offspring [13]. Second, allele severity: the variants that permit male survival, and which are therefore the variants observed in a male-ascertained series such as this one, are enriched for hypomorphic alleles whose heterozygous effect is correspondingly mild; in reported p.Ala152Val (e2 p.Ala140Val) families, carrier females range from unaffected to mildly impaired in cognition and speech [10, 15]. The clinically affected but untested mother of MECP2-013 sits at the recognised mild end of this heterozygous range. X-inactivation studies were not performed in the carrier mothers of this series, so the relative contribution of the two mechanisms in these families is not established.

### An exploratory observation on reading frame

Work on C-terminal frameshift variants has shown that the resulting reading frame, and not only the position of the deletion, influences the consequence, with different frames producing different downstream peptides before termination [16, 17]. This series contains one pair of adjacent-codon variants, MECP2-004 (c.1194_1203del, p.Pro399Hisfs*19; e2 c.1158_1167del) and MECP2-022 (c.1198_1202del, p.Pro400Serfs*3; e2 c.1162_1166del). In that work, deletions of 3n + 2 nucleotides are associated with Rett syndrome and end in a proline-proline-stop motif, while deletions of 3n + 1 nucleotides are associated with benign outcome and end in -SPRTX. Translation of the two transcripts places MECP2-004 in the 3n + 1 class, with the product ending -SPRTX, and MECP2-022 in the 3n + 2 class, although its product ends -PSTX and does not carry the motif itself. The variant in MECP2-004 is the one exception to that association reported in the study: it is modelled there as CTD3, and hemizygous knock-in mice have wild-type MeCP2 protein and transcript levels, no Rett-like phenotype and full survival, from which the authors predict that the two published cases of classical Rett syndrome with this de novo variant are not explained by it. The specification restricts BS3 to RNA studies, so these data could not be applied as a benign criterion, and under the specification the variant is pathogenic on case-level evidence, PVS1, PS4_Supporting and PM2_Supporting (Results). For this variant the classification and the functional data therefore point in opposite directions. We report the specification’s classification, as for every other variant in the series; for counselling we would present it together with the in vivo evidence and with the proband’s reported CACNA1A finding. Because the classification of MECP2-004 rests on case-level evidence and not on reading frame, the pair is not a test of the model, and the difference in clinical picture between the two probands, mild intellectual disability with autism and no seizures in one and an adult-onset parkinsonian course in the other, is not evidence for or against it.

We present this as an exploratory observation and nothing more, for three reasons. First, it rests on a single pair. Second, the classification of one member of that pair rests on case-level evidence and that of the other on its absence, so neither can serve as a control for the other. Third, and importantly, the published model concerns the C-terminal deletion-prone region, approximately c.1110 to c.1210 in e2 numbering (c.1146 to c.1246 in MeCP2_e1), and two of our five frameshift variants fall outside it: MECP2-019 (c.1334del) lies downstream of the region, and MECP2-015 (c.1122dup) lies upstream. MECP2-015, whose variant is of uncertain significance, has a severe presentation with a refractory Lennox-Gastaut course, which we bring forward explicitly as a qualifier, while noting that it lies outside the region and that neither its classification nor the evidence available for it constitutes direct evidence against the model. No functional work was performed on the variants of this series. The observation played no part in our classifications: PVS1 strength was assigned solely by the specification’s MECP2-specific rule and was not modulated by frame.

### p.Ala152Val

p.Ala152Val (e2 p.Ala140Val) is a long-recognised hypomorphic allele with variable and reduced penetrance [10, 12, 15]. It was the most frequent variant in this series, in four unrelated probands, and the phenotypes among the four differed considerably, from mild disability with a happy demeanour to severe motor involvement with autism and self-directed harm. The mother of one of the four is clinically affected with mild intellectual disability, although molecular confirmation could not be obtained. The variant is present in gnomAD v4.1.0, including one hemizygous individual, which is why PM2 is not applicable and why, under the specification, PS4 cannot be used despite the recurrence in this series; the classification rests instead on PM1, PP3 and co-segregation in two published families [12, 15]. Apart from this study’s own submission, ClinVar holds 35 submissions with a classification for this allele, all pathogenic or likely pathogenic and none from the Expert Panel (ClinVar 11823). The difference between that aggregate and our likely pathogenic classification follows from the gene-specific instruction, which withholds the case-count criterion from variants present in population data, and not from a different reading of the published disease associations. We consider that this presence does not argue against a role in disease. gnomAD excludes severe paediatric disease but does not exclude mild neurodevelopmental or psychiatric phenotypes, so a hemizygous adult in that dataset is evidence against a fully penetrant severe phenotype rather than evidence of clinical normality. The classification is likely pathogenic, but for counselling we would describe the variant as a hypomorphic allele with reduced penetrance rather than as a straightforward likely pathogenic finding, since the two convey different expectations to a family.

### The 3’ untranslated region deletion

The deletion in MECP2-016 removes no coding nucleotide and lies wholly within the 3’ untranslated region. It is nevertheless not an arbitrary interval: on the coordinates reported by the referring laboratory, it contains the annotated 3’ end of the short-3’UTR MECP2 transcript and two canonical polyadenylation hexamers. MECP2 uses alternative polyadenylation, and the long 3’ untranslated region is a substrate for microRNA-mediated regulation, in a gene whose dosage sensitivity is well established in both directions. A shift in the balance between the short and long 3’UTR isoforms is therefore a mechanism that cannot be excluded. We state plainly that this is speculation. No RNA was available from this proband, isoform ratios were not measured, the deletion itself has not been confirmed by an independent method, and its breakpoints are approximate. The variant is classified as of uncertain significance, and no criterion, pathogenic or benign, was applied.

### Interpretation in MECP2 in males

Two general points follow from the reclassification. First, applying a gene-specific expert specification moved this series away from pathogenicity rather than towards it. Under the specification, 13 of the 17 distinct variants are of uncertain significance and 1 is benign, which corresponds to 14 of the 22 probands and 1 of the 22 probands respectively. The effect is most marked among the truncating variants: all five frameshift variants in this series meet PVS1 at Very Strong, yet only one reached pathogenic, on case-level evidence for the identical variant, and the other four are of uncertain significance. A predicted loss-of-function variant in MECP2 does not carry case-level evidence with it, and in a gene of this size the number of laboratories asserting pathogenicity in a public database is not a substitute for that evidence. Second, population data must be used with care in X-linked genes. Hemizygote counts on the X chromosome are subject to technical artefact, including errors of sex inference and variable coverage, and a single hemizygote in a population database should not by itself settle a classification in either direction; the specification’s own instruction to reserve BS2 for phenotyped individuals reflects the same caution.

### Limitations

This series has substantial limitations, and the observations above should be read in their light.

Ascertainment through clinical genetics centres selects for individuals in whom testing was thought worthwhile, so the series is not representative of MECP2-related disease in males in the population. The testing indications, which include unexplained epilepsy and developmental delay, shape the phenotypic distribution directly.

Phenotyping was retrospective and not uniform. Data were extracted from diagnostic records written for clinical rather than research purposes; the depth of recording varied between centres and between probands; and denominators therefore differ between headings. Exact ages at the last assessment could be retrieved for only seven probands, and several outcomes reported here, including obesity, epilepsy and neuropsychiatric features, are age-dependent, so the denominators for these outcomes include children who may not yet have reached the age of risk. Growth and head-circumference findings are clinician-recorded observations without uniform definitions, cut-offs or reference charts, and underlying measurements and standard deviation scores could not be retrieved; the growth counts reported here should be read accordingly, and for one proband a raw body mass index recorded in childhood could not be converted to a standard deviation score. Per-proband neuroimaging availability could not be reconstructed, so imaging findings are reported without a denominator. Dysmorphology assessment was not performed uniformly and is not tabulated. Missing data were not imputed.

Fourteen of the 21 probands in the analysis cohort carry variants of uncertain significance. For these probands, causation has not been established, and their inclusion in a description of the clinical range is a decision that a reader may reasonably question. We have separated them from the pathogenic and likely pathogenic group throughout so that the basis of each statement is visible. The pathogenic and likely pathogenic group is correspondingly small, and four of its seven probands carry the same recurrent p.Ala152Val allele, so the phenotypic range that can be attributed to MECP2 in this series is narrower than the size of the ascertained series suggests. The classification of MECP2-004 in this group rests on case-level evidence under the specification, whereas the only functional study of the identical variant predicts it to be benign, and that proband also carries a reported CACNA1A variant. MECP2-010, whose extreme prematurity with hypoxic-ischaemic encephalopathy is a sufficient explanation for his phenotype on its own, contributes to the counts of the uncertain group, and key growth counts are therefore also given with this proband excluded.

No functional studies were performed on the variants of this series. The reading-frame observation rests on one pair of variants. The regulatory hypothesis for the 3’UTR deletion in MECP2-016 has no supporting data at all, and the deletion itself was identified by exome-based copy number analysis, was not confirmed by an independent method, its calling pipeline was not recorded, and its breakpoints are approximate. Chromosome analysis and microarray were normal in that proband but cannot resolve an event of this size.

Runs of homozygosity were not analysed and no systematic search for a second recessive diagnosis was made beyond the diagnostic pipelines, although two families are consanguineous; the multi-locus observations therefore rest on incidentally detected findings. For those findings, complete variant nomenclature with transcript is available only for the IGSF1 variant, no ACMG/AMP class is recorded for the PGM3 and IGSF1 findings, and no formal segregation study was available for any of them, so they could not be independently reclassified for this study and should not be read as confirmed secondary findings. Formal kinship analysis was not performed for the four p.Ala152Val probands, who originate from different provinces and have no known relatedness, or for the two p.Arg179Trp probands; a shared founder haplotype cannot be excluded, and the recurrence counts should be read with that caveat.

A maternal carrier was identified in only 6 of 22 families, in one of them on the referring clinician’s documentation rather than a retrievable laboratory record, so no inference about de novo events can be drawn. The affected maternal uncle of MECP2-021 was not genotyped because the family declined testing, so co-segregation in that family rests on clinical history alone and PP1 was not applied. The clinically affected mother of MECP2-013, the most informative untested individual in the series, could not be tested for this study. Sanger chromatogram images were not retrievable from the diagnostic archives of the participating centres, and the segregation data reported here rest on the diagnostic reports. The consent obtained does not cover the publication of identifiable images, so craniofacial findings are not reported photographically, which limits their comparability with published series. One proband has died, and his data derive from records alone.

Finally, the series is small. Subgroups defined by protein domain would contain too few probands to support any claim of genotype-phenotype correlation, and we have therefore not attempted such an analysis.

## Conclusions

In a series ascertained through routine diagnostic testing rather than through severe-phenotype registries, the phenotypes observed among hemizygous males carrying MECP2 variants were graded rather than uniformly severe. Among the probands with pathogenic or likely pathogenic variants they spanned mild to severe syndromic intellectual disability with autism-spectrum and epilepsy presentations, and one man carrying a variant of uncertain significance had an adult-onset parkinsonian course for which causation was not established. Growth or head-circumference findings were recorded for only 9 of the 21 probands in the analysis cohort; among these nine, obesity or overgrowth was recorded in 5, microcephaly in 4 and short stature in 3. These are counts from unevenly phenotyped records and they support no comparative claim.

These observations are provisional. Fourteen of the 21 probands in the analysis cohort carry variants of uncertain significance, and the range that may be attributed to MECP2 rests on seven individuals carrying variants classified as pathogenic or likely pathogenic under the stated criteria. In one of them, MECP2-004, the pathogenic classification rests on case-level evidence, whereas the only functional study of the identical variant predicts it to be benign, and the proband also carries a reported CACNA1A variant. Attribution of his phenotype to MECP2 therefore remains uncertain. Reclassification under the ClinGen Expert Panel specification moved the series away from pathogenicity, and in our hands the specification, applied with its own combining rules, gave classifications that could be audited criterion by criterion; we found it well suited to MECP2 variant assessment in males.

Two practical points emerge, offered as observations from this series rather than as established recommendations. A low threshold for MECP2 testing in males with unexplained neurodevelopmental presentations appears warranted, since these probands were found through indications that made no reference to MECP2. Growth and metabolic parameters merit attention alongside head circumference. Where a second candidate locus is reported, as in 3 of our 22 probands, the MECP2 variant should not be assumed to account for the whole phenotype. Larger series with uniform phenotyping, and functional work on C-terminal and non-coding variants, will be needed to place any of this on a firmer footing.

## Supplementary information

Below is the link to the electronic supplementary material.

Additional file 1

Additional file 2

## Data Availability

The de-identified, proband-level dataset supporting the conclusions of this article is included within the article and its additional file (Additional file 1: Table S1). Any further details are available from the corresponding author on reasonable request. Variants identified in this study have been submitted to ClinVar (https://www.ncbi.nlm.nih.gov/clinvar/; Submission ID: SUB16291306); accession numbers (SCV) will be added upon release by ClinVar and are available from the corresponding author in the interim.

## Abbreviations

ACMG/AMP: American College of Medical Genetics and Genomics/Association for Molecular Pathology
ACTH: adrenocorticotropic hormone
ADHD: attention-deficit/hyperactivity disorder
ASD: autism spectrum disorder
ASM: antiseizure medication
BS1: benign strong criterion 1
BS2: benign strong criterion 2
ClinGen: Clinical Genome Resource
CNV: copy-number variant
CTD: C-terminal domain
DD: developmental delay
EEG: electroencephalography
FAF: filtering allele frequency
gnomAD: Genome Aggregation Database
HGVS: Human Genome Variation Society
ID: intellectual disability
MANE: Matched Annotation from NCBI and EMBL-EBI
MBD: methyl-CpG-binding domain
MDS: MECP2 duplication syndrome
MeCP2: methyl-CpG-binding protein 2
MRI: magnetic resonance imaging
NGS: next-generation sequencing
NID: NCoR/SMRT interaction domain
OCD: obsessive-compulsive disorder
OFC: occipitofrontal circumference
PAS: polyadenylation signal
PPM-X: pyramidal signs, parkinsonism and macroorchidism (X-linked)
PS4: pathogenic strong criterion 4
PVS1: pathogenic very strong criterion 1
SDS: standard deviation score
TRD: transcriptional repression domain
UTR: untranslated region
VCEP: Variant Curation Expert Panel
VUS: variant of uncertain significance
WES: whole-exome sequencing
