# Supplementary material for: The clinical spectrum of MECP2 variants in males: a multicenter Turkish cohort of 22 probands, with implications for diagnosis and variant interpretation
Source: BMC Pediatr. 2026 Oct 21;26:1056. doi: 10.1186/s12887-026-07905-0 (PMC13693129; doi:10.1186/s12887-026-07905-0)
Supplement: Supplementary file 1 [file 12887_2026_7905_MOESM2_ESM.docx]

**STROBE Statement: checklist of items that should be included in reports of observational studies**

Manuscript: "The clinical spectrum of MECP2 variants in males: a multicenter Turkish cohort of 22 probands, with implications for diagnosis and variant interpretation".

Design and column used: the study is a multicentre, retrospective, record-based series of probands assessed at a single time point, with no comparison group and no defined follow-up period. The cross-sectional column of the combined STROBE checklist is therefore the closest fit and has been used throughout. Items that apply only to cohort or case-control designs are marked as not applicable, with the reason given.

Locations are given by section and subsection of the revised manuscript rather than by line number, since these remain stable if the text repaginates; the manuscript file also carries continuous line numbers for reviewer reference.

| **Section / topic** | **Item No** | **Recommendation** | **Location in the revised manuscript** |
| --- | --- | --- | --- |
| Title and abstract | 1a | Indicate the study design with a commonly used term in the title or the abstract | Title ("a multicenter Turkish cohort of 22 probands"); Abstract, Methods, first sentence (multicentre series ascertained through routine diagnostic testing) |
|  | 1b | Provide in the abstract an informative and balanced summary of what was done and what was found | Abstract, Background through Conclusions (structured) |
| **Introduction** |  |  |  |
| Background / rationale | 2 | Explain the scientific background and rationale for the investigation being reported | Background, paragraphs 1 to 3 |
| Objectives | 3 | State specific objectives, including any prespecified hypotheses | Background, final paragraph (three stated aims; no prespecified hypothesis) |
| **Methods** |  |  |  |
| Study design | 4 | Present key elements of study design early in the paper | Methods, Ascertainment and testing indications, paragraph 1; Methods, Cohort structure |
| Setting | 5 | Describe the setting, locations, and relevant dates, including periods of recruitment, exposure, follow-up, and data collection | Methods, Ethics approval and consent (Ordu University Clinical Research Ethics Committee, approval no. 2026/19, 23 January 2026); Methods, Ascertainment and testing indications (retrospective identification from the diagnostic records of participating medical genetics centres across Turkiye). A calendar period for assembly of the diagnostic records is not stated in the manuscript, as the source records were compiled retrospectively across centres and a common start and end date could not be established |
| Participants | 6a | Give the eligibility criteria, and the sources and methods of selection of participants | Methods, Ascertainment and testing indications (testing indications; inclusion criteria in the final sentence: male sex, hemizygosity for a MECP2 variant, records sufficient for the fields reported) |
|  | 6b | Cohort and case-control studies only: matching criteria and numbers of exposed and unexposed / cases and controls | Not applicable. This is a single-group descriptive series with no comparison group and no matching |
| Variables | 7 | Clearly define all outcomes, exposures, predictors, potential confounders, and effect modifiers. Give diagnostic criteria, if applicable | Methods, Variant nomenclature and coordinates; Methods, Variant classification (ClinGen GN036 v6.0.0 criteria, thresholds and combining rules); Methods, Phenotypic data and handling of missing data |
| Data sources / measurement | 8 | For each variable of interest, give sources of data and details of methods of assessment. Describe comparability of assessment methods if there is more than one group | Methods, Molecular analysis (sequencing performed at referring centres under local protocols; diagnostic reports as source documents); Methods, Phenotypic data and handling of missing data (retrospective extraction from diagnostic records). Comparability is addressed in Discussion, Limitations, paragraph 2 (no uniform phenotyping protocol; depth of recording varied between centres) |
| Bias | 9 | Describe any efforts to address potential sources of bias | Methods, Phenotypic data and handling of missing data (no imputation; per-variable denominators); Methods, Cohort structure (the benign proband excluded from all phenotypic denominators); Discussion, Limitations, paragraphs 1 and 2 (ascertainment through diagnostic centres; indication-driven phenotype distribution) |
| Study size | 10 | Explain how the study size was arrived at | Methods, Cohort structure; Results, Variant spectrum, paragraph 1. The size was determined by the number of hemizygous males identified through routine diagnostic testing at the participating centres (22 ascertained, 21 in the analysis cohort); no a priori sample size calculation was performed |
| Quantitative variables | 11 | Explain how quantitative variables were handled in the analyses. If applicable, describe which groupings were chosen and why | Methods, Phenotypic data and handling of missing data (ages reported as recorded; growth and head-circumference findings treated as clinician-recorded observations, not standardised anthropometry); Methods, Statistical analysis; Methods, Cohort structure (grouping by final variant classification) |
| Statistical methods | 12a | Describe all statistical methods, including those used to control for confounding | Methods, Statistical analysis (descriptive throughout; no formal statistical comparison; no adjustment for confounding was attempted) |
|  | 12b | Describe any methods used to examine subgroups and interactions | Methods, Cohort structure (two prespecified groups, pathogenic or likely pathogenic and uncertain, reported separately); Results, clinical findings sections. No interaction analysis was performed |
|  | 12c | Explain how missing data were addressed | Methods, Phenotypic data and handling of missing data (missing values not imputed; fields without a record marked as not recorded and excluded from the corresponding denominator); Methods, Statistical analysis |
|  | 12d | Cross-sectional studies: if applicable, describe analytical methods taking account of sampling strategy | Not applicable. There was no sampling strategy; all eligible probands identified at the participating centres are included |
|  | 12e | Describe any sensitivity analyses | Results, Growth and head circumference across the analysis cohort (key counts given with and without MECP2-010, whose extreme prematurity with hypoxic-ischaemic encephalopathy is a sufficient alternative explanation) |
| **Results** |  |  |  |
| Participants | 13a | Report numbers of individuals at each stage of the study | Results, Variant spectrum, paragraph 1; Results, Classification; Methods, Cohort structure (22 ascertained; 21 in the analysis cohort; 1 reported separately) |
|  | 13b | Give reasons for non-participation at each stage | Results, Inheritance and segregation (mothers of 16 probands not tested; the family of MECP2-021 declined testing of the affected maternal uncle; the mother of MECP2-013 could not be tested for this study); Discussion, Limitations |
|  | 13c | Consider use of a flow diagram | Not included. The series has two stages only, 22 ascertained and 21 analysed, and the numbers with the reason for the difference are stated in Methods, Cohort structure and in Results, Classification |
| Descriptive data | 14a | Give characteristics of study participants and information on exposures and potential confounders | Results, clinical findings sections; Table 3; Additional file 1, Table S1b; Results, Secondary findings and Table 4 (second candidate loci) |
|  | 14b | Indicate number of participants with missing data for each variable of interest | Results, clinical findings sections (every proportion given as n/N); Table 3 and Additional file 1, Table S1b (not recorded marked per cell); Methods, Phenotypic data and handling of missing data |
|  | 14c | Cohort studies: summarise follow-up time | Not applicable. Data derive from the most recent documented assessment; no systematic follow-up period was defined or recorded |
| Outcome data | 15 | Report numbers of outcome events or summary measures | Results, clinical findings sections; Results, Growth and head circumference across the analysis cohort; Results, Inheritance and segregation; Tables 1, 2 and 3 |
| Main results | 16a | Give unadjusted estimates and, if applicable, confounder-adjusted estimates and their precision. Make clear which confounders were adjusted for and why they were included | Results, Classification and clinical findings sections. All results are unadjusted descriptive counts and proportions with explicit denominators; no modelling was performed, so no adjusted estimates or confidence intervals are reported |
|  | 16b | Report category boundaries when continuous variables were categorized | Results, clinical findings sections (severity grades as recorded; borderline microcephaly identified as such wherever counted); Methods, Phenotypic data and handling of missing data (no uniform cut-offs or growth references could be retrieved, so categories are those recorded by the referring clinicians) |
|  | 16c | If relevant, consider translating estimates of relative risk into absolute risk for a meaningful time period | Not applicable. No relative risk estimates were calculated |
| Other analyses | 17 | Report other analyses done, for example analyses of subgroups and interactions, and sensitivity analyses | Results, Secondary findings; Results, Variant classified as benign; Discussion, An exploratory observation on reading frame; Discussion, p.Ala152Val |
| **Discussion** |  |  |  |
| Key results | 18 | Summarise key results with reference to study objectives | Discussion, opening paragraph; Conclusions |
| Limitations | 19 | Discuss limitations of the study, taking into account sources of potential bias or imprecision. Discuss both direction and magnitude of any potential bias | Discussion, Limitations (entire subsection: ascertainment, non-uniform retrospective phenotyping, missing ages and growth measurements, preponderance of uncertain variants, absence of functional and runs-of-homozygosity analyses, incomplete maternal genotyping, small size) |
| Interpretation | 20 | Give a cautious overall interpretation of results considering objectives, limitations, multiplicity of analyses, results from similar studies, and other relevant evidence | Discussion, all subsections; Conclusions (findings stated as provisional and resting on seven probands) |
| Generalisability | 21 | Discuss the generalisability (external validity) of the study results | Discussion, paragraphs 1 and 2; Discussion, Limitations, paragraph 1; Table 5 and its footnote (ascertainment differences preclude direct comparison with published series) |
| **Other information** |  |  |  |
| Funding | 22 | Give the source of funding and the role of the funders for the present study and, if applicable, for the original study on which the present article is based | Declarations, Funding (no external funding) |

An Explanation and Elaboration article discusses each checklist item and gives methodological background and published examples of transparent reporting. The STROBE checklist is best used together with that article, which is freely available at https://www.strobe-statement.org.
